# The Efficacy of Fixed-Dose Diclofenac and Orphenadrine for Postoperative Pain Management: A Systematic Review

**DOI:** 10.3390/medicines13020017

**Published:** 2026-05-08

**Authors:** Nikolaos Christopoulos, Karolina Akinosoglou

**Affiliations:** 1 School of Science and Technology, Hellenic Open University, 18 Aristotelous St., 26335 Patras, Greece; nchristop.ph@gmail.com; 2 Department of Medicine, University of Patras, Rio, 26504 Patras, Greece; 3 Department of Internal Medicine and Infectious Diseases, University General Hospital of Patras, Rio, 26504 Patras, Greece

**Keywords:** diclofenac, orphenadrine, postoperative, pain, opioids, analgesia

## Abstract

Background/Objectives: Postoperative pain remains a significant clinical challenge, often requiring multimodal strategies to mitigate opioid-related adverse events. The fixed-dose combination (FDC) of Diclofenac, a non-steroidal anti-inflammatory drug, and Orphenadrine, a muscle relaxant, targets distinct nociceptive pathways to potentially enhance analgesia and reduce opioid consumption. This systematic review aims to evaluate the analgesic efficacy and safety profile of the fixed-dose combination of Diclofenac and Orphenadrine for postoperative pain management and quantify its opioid-sparing effect compared to standard monotherapies or placebo. Methods: A systematic search of electronic databases (MEDLINE, Scopus) and clinical trial registries (including ClinicalTrials.gov and CTIS) was conducted up to 20 September 2025. Fourteen (14) randomized controlled trials (RCTs) involving 981 adult patients undergoing various surgical procedures were included. Due to high clinical and methodological heterogeneity, a Synthesis Without Meta-analysis (SWiM) approach was utilized. The certainty of evidence was assessed using the GRADE methodology. Results: The synthesis demonstrated that the FDC may improve pain relief (measured by the Visual Analog Scale and Numeric Rating Scale scores) and may reduce opioid consumption compared to active comparators and placebo. The opioid-sparing effect could be correlated with a reduced incidence of dose-dependent adverse events, particularly nausea and vomiting. However, the overall certainty of the evidence was graded as “Very Low” due to the high risk of bias and lack of transparency in the included studies. Conclusions: The FDC of Diclofenac and Orphenadrine is a rational addition to multimodal postoperative analgesic regimens, which may potentially reduce the perioperative opioid burden without compromising pain control. Nevertheless, because almost all included studies suffer from severe methodological flaws, these apparent efficacy findings must be interpreted with caution. Future high-quality, pre-registered, and low-bias randomized controlled trials are required to draw firm clinical conclusions.

## 1. Introduction

Despite significant advancements in surgical techniques and anesthetic protocols, postoperative pain remains prevalent and inadequately managed in the clinical setting. Current literature suggests that despite the widespread implementation of acute pain protocols, a substantial proportion of patients undergoing various surgery procedures report moderate to severe pain in the immediate postoperative period [1].

Uncontrolled acute pain is not merely a patient comfort issue since it activates the sympathetic nervous system [2], increases cardiac demand, impairs pulmonary function [3], and delays mobilization, thereby hindering Enhanced Recovery After Surgery (ERAS) protocols [4,5]. Furthermore, the transition from acute to chronic postsurgical pain is a complex pathophysiological process driven primarily by sustained peripheral and central sensitization [6,7]. When intense nociceptive stimuli persist due to surgical trauma, the release of local inflammatory mediators lowers the activation threshold of peripheral nociceptors [6]. This continuous barrage of peripheral noxious signaling triggers enduring, maladaptive neuroplastic changes within the central nervous system [8]. Specifically, the activation of N-methyl-D-aspartate (NMDA) receptors in the spinal dorsal horn and subsequent neuroimmune interactions, including microglial activation, sustain pain sensation long after normal tissue healing is complete [9]. Because these structural and biochemical shifts establish an intractable chronic pain state, aggressive and effective early multimodal pain control can intercept this chronification process [7].

Historically, the primary pharmacological approach to intercepting this pain relied heavily on opioids, but surgery has been identified as a distinct gateway to chronic opioid use, with incidence rates of new persistent use ranging from 1.4% to 6.5% in opioid-naive patients, and up to 13% in the general surgical population [10]. Consequently, the modern paradigm of perioperative care has shifted decisively toward opioid-sparing analgesia to mitigate opioid-related adverse events such as respiratory depression, ileus, and postoperative nausea and vomiting (PONV) [11].

To address these challenges, major medical societies advocate for a move away from opioid monotherapy. Collaborative guidelines released in 2016 by the American Pain Society (APS), the American Society of Anesthesiologists (ASA), and the American Society of Regional Anesthesia (ASRA) identify multimodal analgesia as the foundational strategy for effective postoperative pain control [12]. The guidelines recommend combining pharmacological interventions with distinct mechanisms of action alongside adjuvants to maximize pain relief while minimizing opioid consumption and associated adverse events [12]. 

The procedure-specific pain management (PROSPECT) collaboration emphasizes that optimal analgesia must be procedure-specific, as the efficacy of analgesic combinations varies significantly depending on the surgical site and tissue trauma involved [13]. 

The rationale for combining Diclofenac and Orphenadrine lies in the potential for additive or synergistic analgesia through concurrent targeting of different nociceptive pathways. Diclofenac, a potent NSAID, exerts its effect primarily through the inhibition of cyclooxygenase (COX-1 and COX-2) enzymes, thereby reducing the synthesis of pro-inflammatory prostaglandins that sensitize peripheral nociceptors [14]. Orphenadrine primarily acts within the central nervous system, blocking facilitation of motor reflexes at the spinal and supraspinal levels. It inhibits the facilitation of the knee jerk and relieves decerebrate rigidity, suggesting a central site of action distinct from direct neuromuscular blockade [15]. This central effect is not solely due to its anticholinergic or antihistaminic properties, but rather involves interference with reticulospinal pathways that modulate muscle tone and reflexes [15]. While its precise mechanism involves anticholinergic activity, it is also identified as a non-competitive N-methyl-D-aspartate (NMDA) receptor antagonist [16]. Therefore, the fixed-dose combination (FDC) aims to target both the peripheral inflammatory cascade and central muscular/neuropathic mechanisms. Diclofenac covers the analgesic and anti-inflammatory effect, whereas Orphenadrine works as an adjuvant for pain and muscle spasm, symptoms known to appear in the postoperative setting [17]. 

Clinical interest in this specific combination has grown in specific regions. The Russian Association for the Study of Pain (RASP) in 2024 stated that the fixed infusion combination of Orphenadrine (30 mg) and Diclofenac (75 mg) has demonstrated high efficacy and safety in treating acute spondylogenic and acute non-specific low back pain. The experts recommended including this combination in updated clinical guidelines for the management of acute non-specific low back pain [18]. Additionally, in a review by Sorokina et al., the fixed intravenous combination of Diclofenac (75 mg) and Orphenadrine (30 mg) was described as effective in multimodal analgesia for moderate-to-severe postoperative pain across cardiac, thoracic, abdominal, and orthopedic surgeries. The authors conclude that this combination significantly reduces opioid consumption (demonstrating a marked opioid-sparing effect) [19]. These findings were echoed by the 2023 Expert Council Resolution summarizing the NEODOLEX-S observational studies, which highlighted the combination’s high safety profile and distinct opioid-sparing capabilities in the early postoperative period [20]. Despite these findings, the regulatory and commercial landscape for this combination varies. Not many Diclofenac-containing fixed-dose products exist currently in the EU market; those that do are mostly combinations with thiocolchicoside or tribenoside. Specifically for Diclofenac and Orphenadrine, the primary formulation is Neodolpasse (Fresenius Kabi), administered as an injection indicated for acute pain syndrome and postoperative pain. While fixed-dose combinations (FDCs) are generally beneficial for older adults by simplifying dosing regimens and improving adherence, caution is required. Orphenadrine-containing FDCs are generally avoided in this population [21].

While recent systematic reviews evaluating other Diclofenac + muscle relaxant combinations found that the combination can be an effective option to combat pain [22], there is no clear superiority of Diclofenac/Orphenadrine combination over other monotherapies. Most existing data are derived from observational studies or regional expert consensus rather than a high-quality synthesis of Randomized Controlled Trials (RCTs). Therefore, the aim of this systematic review is to compare the efficacy of the Diclofenac and Orphenadrine combination for postoperative pain and quantify its opioid-sparing effect compared to standard treatment monotherapies or placebo. Reviewing current evidence is crucial to consolidate findings regarding its analgesic superiority and safety profile to inform evidence-based postoperative pain guidelines.

## 2. Materials and Methods

### 2.1. Inclusion and Exclusion Criteria

Evidence from interventional studies was included in this systematic review. The inclusion of randomized controlled trials aimed to assess the efficacy and safety profile of the combination therapy and minimize the risk of bias. Only original research data published in scientific journals were eligible for inclusion; abstracts without full text available were excluded from the analysis to preserve the quality of the evidence. No language-specific criteria for the publications were used. 

To be included, studies had to be randomized controlled trials that enrolled adults (18 years of age or older) treated with Diclofenac and/or Orphenadrine for post-operative pain after various surgical procedures. The intervention of interest was the fixed combination of Diclofenac with Orphenadrine, regardless of the dosage or the route of administration. Eligible comparator groups included monotherapy with Diclofenac or other NSAIDs, alternative treatments for post-operative pain, and/or placebo. Primary outcome measures included tools for pain relief measurement, like the VAS (Visual Analog Scale) and NRS (Numeric Rating Scale), to determine pain intensity and duration of analgesia, as well as the Opioid Sparing Effect. Secondary outcome measures included the incidence of adverse events reported to address the combinations’ safety. 

This systematic review was conducted and reported in accordance with the PRISMA 2020 guidelines [23]. The completed PRISMA checklist is available as Supplementary File S1. A PICO table summarizing the eligibility criteria for the randomized controlled trials is presented below (Table 1). 

### 2.2. Information Sources

To identify eligible studies, we conducted a search of electronic databases and clinical trial registries. The bibliographic databases included MEDLINE (via PubMed), Scopus, and ResearchGate. For the Scopus search, results were limited to the subject areas of medicine, pharmacology, toxicology, pharmaceutics, health professions, dentistry, and neuroscience. To locate ongoing or unpublished trials and match publications with their protocols, we also searched ClinicalTrials.gov, the EU Clinical Trials Information System (CTIS), the older version of the EU Clinical Registry, and the WHO trials registry. No language restrictions were applied to our search. The run for the databases and registries included all records up to 20 September 2025. While a formal review procedure was followed internally to guide the methodology, it was not prospectively registered prior to study initiation. However, to maximize transparency and reproducibility, the study protocol and methodological details have since been retrospectively deposited on the Open Science Framework (OSF) and can be accessed at osf.io/qmvtn.

### 2.3. Search Strategy

A literature search was executed to identify studies assessing the combined use of Diclofenac with Orphenadrine for managing post-operative analgesia. The search included publications up to 20 September 2025. To specifically target studies addressing both therapies and their combined use, the keywords “Diclofenac” and “Orphenadrine” were coupled with “AND”. The search strategy was intentionally focused on the two generic drug names to ensure high specificity. No natural language processing, text analysis tools, or previously published search filters were used, and the strategy was not peer reviewed. The rationale for this strategy was to retrieve all studies where both Diclofenac and Orphenadrine were explicitly mentioned. In our process, we relied on a manual screening of all retrieved records to identify eligible sources concerning post-operative pain, rather than beginning with a more sensitive but less specific search that would include numerous synonyms and condition-related terms. The full, reproducible search syntax for each database and registry, including any applied limits, is provided in the Supplementary Material S3.

### 2.4. Study Selection Process

All records retrieved from the search of the bibliographic databases and trial registries, as described earlier, were stored and deduplicated using Zotero (v7.0.32), and subsequently screened independently by two reviewers. While a formal pilot calibration exercise was not conducted, both reviewers thoroughly discussed the eligibility criteria prior to initiating the screening process to ensure consistent interpretation. Initially, the title and abstract of each record were compared against the eligibility criteria. Records were excluded only if they were clearly irrelevant. If no minimal essential information was collected from the abstract, we proceeded with the retrieval of the full text. Disagreements between the reviewers were thoroughly discussed until a consensus was reached. We did not use any automation tools or crowdsourcing for the screening process; all records were manually assessed for eligibility. Reports not meeting the inclusion criteria were documented, along with the primary reason for exclusion. If papers were not in English, a translation was performed using tools such as DocTranslator (https://www.onlinedoctranslator.com/en/, accessed on 27 April 2026) and DeepL (v26.2.1) to assess eligibility. These translated texts were subsequently reviewed and verified by the human reviewers to ensure the accurate interpretation of clinical and methodological data prior to determining final eligibility.

### 2.5. Data Collection Process and Data Items

The data collection process included a standard electronic data extraction spreadsheet via Microsoft Excel, which was securely stored on a shared drive to maintain data integrity and accessibility among the reviewers. This process was conducted independently by both reviewers to minimize any selection bias, with all identified disagreements resolved through discussion. The set of data items collected was defined prior to extraction, including basic study design characteristics (e.g., population, intervention, comparisons, outcomes, randomization, and blinding details). Explicit details were collected on efficacy outcomes, including specific measures, specifically the severity of pain assessed by the patients using the Visual Analog Scale (VAS) or the Numeric Rating Scale (NRS), and the consumption of opioids, if used to determine the opioid sparing effect to address the primary outcome of this review. Emphasis was also given to the adverse events reported in the selected studies to determine the combination’s safety. Details on the methodology relevant to internal validity, such as blinding and randomization process, alongside recognized study limitations by the authors and their reported conflict of interest, were systematically extracted. Additionally, numerical data from study graphs were extracted using WebPlotDigitizer if raw data were not reported by the authors.

### 2.6. Quality Assessment and Risk of Bias

To address the bias, the Cochrane handbook was followed [24] using RoB2 for individual RCTs [25] as a validated method to determine the internal validity of the selected studies. The Rob2 tool was applied to assess the risk of bias per outcome across the randomization process, deviations from intended interventions, missing data, measurement of the outcome, and selection of the reported data. For each domain, a judgment of “Low”, “Some concerns”, or “High” was made, and an overall score was given as per this tool’s guidance. Both reviewers thoroughly discussed the RoB2 signaling questions and domain criteria prior to formal assessment. While a formal pilot calibration exercise was not conducted, the bias of all studies was independently assessed by both reviewers, with any disagreements resolved through discussion to reach a consensus without relying on statistical measures of agreement. Since the review investigated pain relief, strict attention was given to domain 4 (D4) to assess the blinding of the outcome assessors; while D4 encompasses multiple methodological elements, trials lacking adequate blinding for this subjective outcome were carefully scrutinized, as the patient’s awareness of their allocated intervention significantly increases the probability of measurement bias. Additionally, for domain 5 (D5: Selection of the reported outcome), studies in which no statistical analysis plan or protocol was publicly available, or those with vague details in their reporting, were judged to be at a high risk of bias. Overall risk-of-bias determinations were made using the standard RoB2 algorithmic approach, where the overall judgment for a result is dictated by the highest risk of bias identified in any single domain. Visualizations of the bias assessments are displayed using the robvis tool.

### 2.7. Effect Measures

Primary outcomes focused on pain intensity and the opioid-sparing effect, utilizing the following metrics to ensure comparability across studies:

1. Pain Intensity:

Assessed using validated patient-reported outcomes such as the Visual Analog Scale (VAS) (typically measured on a 0 to 100 mm scale) and the Numeric Rating Scale (NRS) (typically measured on a 0 to 10 scale). For studies reporting pain scores on different scales (e.g., 0–10 and 0–100), scores were converted to the 0–100 scale for consistency in tabulation and comparison. The standardized metrics chosen for synthesizing pain intensity across the included studies are the mean difference (MD) and median difference (MeD) calculated at specific, individual time points. The selection between MD and MeD for any given study depends on whether the original authors reported means (for normally distributed data) or medians (for non-normally distributed data).

2. Opioid Sparing Effect: This was assessed by two metrics:

- Absolute Opioid Consumption: The mean mg of opioid analgesics consumed over a defined post-operative period. Mean difference is calculated as the metric.
- Opioid Sparing Rate: Defined as the number or proportion of patients who required no postoperative opioid rescue analgesia, or who met a predefined clinical success threshold, measured by risk ratio (RR).

Common adverse events reported in the studies were grouped together via preferred term. The frequency of adverse events was compared between the groups of the selected studies. The effect measure used to compare the frequency of the adverse events between groups was the risk ratio. 

### 2.8. Strategy for Data Synthesis

The constraints presented by substantial clinical and methodological heterogeneity regarding population, dosing regimens, comparators, and inconsistent measurement methods across the included studies precluded a meta-analysis. Consequently, a structured descriptive synthesis approach was adopted to summarize the evidence following the SWiM guideline [26]. The decision to adopt the SWiM approach was made prior to synthesis, immediately after data extraction revealed the extreme clinical and methodological heterogeneity. Specifically, statistical pooling was deemed inappropriate due to the diverse surgical populations evaluated (e.g., mastectomy, orthopedic, cardiac, and dental surgeries), varying dosing regimens, disparate comparators (ranging from inactive placebos to active NSAIDs and strong opioids), and inconsistent outcome measurement time points. According to the Cochrane Handbook, pooling such diverse data into a single meta-analysis would yield a clinically meaningless average effect. The complete SWiM checklist is available in Supplementary Material S2.

Synthesis of pain intensity results was performed using descriptive summaries, structured tabulation, and forest plots (without a pooled summary estimate) of individual study effect estimates. To adhere to SWiM guidelines, studies were grouped for synthesis based on the outcome domain (pain intensity or opioid consumption), the statistical metric reported (means versus medians), and the most common reported postoperative time points in the studies. The rule for judging the direction of effect was based on evaluating whether the individual study effect estimates (mean difference, median difference, or risk ratio) favored the fixed-dose combination or the comparator at each time point, which was visually synthesized using forest plots. To ensure transparency and statistical validity, results from studies reporting means (MD) and medians (MeD) were presented in separate tables and figures and were not merged. These differences were calculated by comparing absolute post-intervention reported scores at the reported time points. In order to compare the mean opioid consumption, the doses of different opioids (e.g., piritramide, tramadol) were converted to equivalent morphine doses for standardization using the eviQ Opioid Conversion Calculator. This metric was measured with the mean difference between the groups. For the percentage of patients not requiring further opioids, the metric used was the risk ratio (RR). Adverse events (AEs) reported across studies were grouped together via preferred term to enable calculation of the risk ratio (RR) based on the extracted data.

For studies that reported the necessary summary statistics (e.g., mean, standard deviation [SD], and sample size [N]) for each intervention group but did not report the 95% confidence interval (95% CI) for the mean difference, the standard error of the difference (SEd) was calculated first and then the 95% CI for the MD was then calculated using accepted methods [24]. The CI for the median difference in studies presented only median values, and only available *p*-values will be presented. The 95% confidence interval for the RR was also computed. Forest plots and spaghetti plots were created using R via Google Colab. Gemini 3.0 pro was used to assist with the generation of the codes for the plots in R.

### 2.9. Assessment of Certainty of Evidence

The overall certainty of the evidence for the outcomes covered in this systematic review (analgesic effect, opioid sparing effect, and adverse events) was systematically assessed using the GRADE methodology. Particular attention was paid to downgrading the certainty of the evidence due to the high risk of bias identified across the included studies (assessed via RoB2), as well as where high inconsistency or indirectness were identified due to high heterogeneity (visually). Given the clinical interdependence of rescue analgesia and pain intensity, the certainty of evidence for pain relief was interpreted in the context of concomitant opioid consumption.

## 3. Results

### 3.1. Study Selection

#### 3.1.1. Flow of Studies

**Figure 1:**
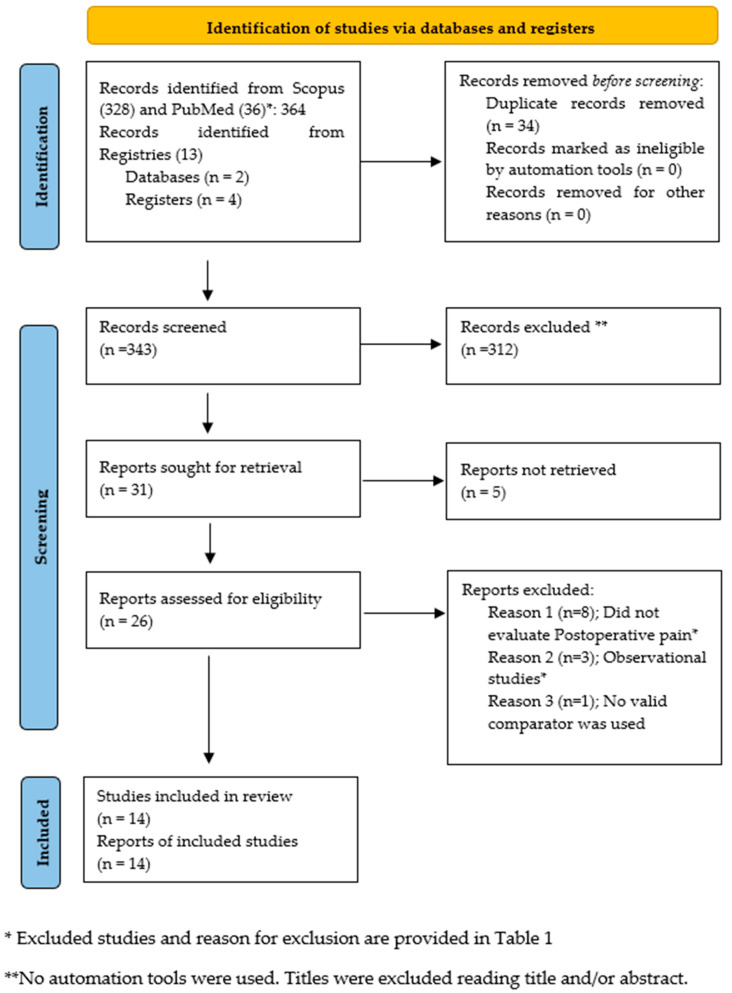
Studies identified using the PRISMA 2020 flow diagram.

From the 377 studies found in registries and databases, 343 records were screened after removing duplicates. Out of these, 312 studies were excluded from the title or abstract cause they were found irrelevant to this systematic review. Thirty-one records were sought for retrieval, but five could not able to be retrieved. Finally, 26 records were assessed for eligibility, with 14 meeting the inclusion criteria [27,28,29,30,31,32,33,34,35,36,37,38,39,40]. It is important to note that the study by Lukonina et al. [40] was characterized in the article as non-interventional; however, since it follows the design of an interventional study, we included it in the analysis. Additionally, the study by Malek et al. [33] was included, but the full text of the journal was not retrievable, and the assessment was made on a publicly available, unpublished thesis (gray literature). The flowchart as per PRISMA guidelines is depicted in Figure 1.

#### 3.1.2. Excluded Studies

The table below (Table 2) provides information on the excluded studies, with the reason for exclusion:

### 3.2. Characteristics of Included Studies

The selected studies included a total of 981 patients after various surgeries (e.g., knee surgeries [33,36,39], mastectomy [27], thoracic surgeries [28,35], cardiac surgeries [30], hip arthroplasty [29,38], abdominal surgeries [32,34], spinal surgeries [40], orthognathic surgeries [37] and other [31]). Approximately 430 patients received Diclofenac+Orphenadrine combination intravenously from all studies combined. Comparator groups included NSAIDs such as Ketorolac+Orphenadrine [27], Ketoprofen [28,34,35,39], Ibuprofen [37,40], Piroxicam [33], Dexketoprofen [32] or monotherapy with morphine [27,29,30,38], tramadol [31,40], pethidine [33], and hydromorphone [36]. In all studies, rescue medication was used, and these included paracetamol [32,37] and several opioids (morphine [27,29,30,35,38,40], pethidine [33], trimeperidine [28,32,39], tramadol [31,34,39,40], hydromorphone [36], and piritramide [37]). Only 4 studies out of the 14 were placebo-controlled [27,29,33,36]. The studies were conducted in various geographical locations, including Russia [28,30,32,34,35,38,39,40], Hungary [31], Austria [29,36,37], Czech Republic [33] and Egypt [27]. All studies evaluated the opioid use, as total opioid consumption or the percentage of patients not requiring any dose. Two studies evaluated pain intensity using the NRS scores [27,37] while the remaining 12 used the VAS [28,29,30,31,32,33,34,35,36,38,39,40]. Only 3 of the selected trials were registered in Clinical Trials databases [29,30,36]. Table 3 and Table 4 below summarize the main study characteristics.

### 3.3. Risk of Bias in Selected Studies

**Figure 2:**
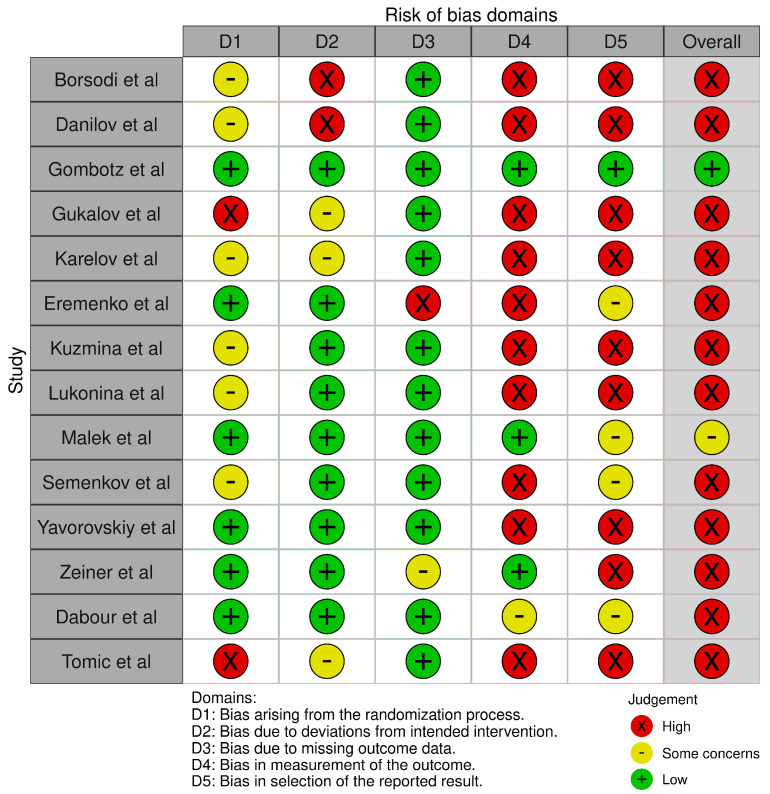
Robvis for pain intensity outcomes [27,28,29,30,31,32,33,34,35,36,37,38,39,40].

**Figure 3:**
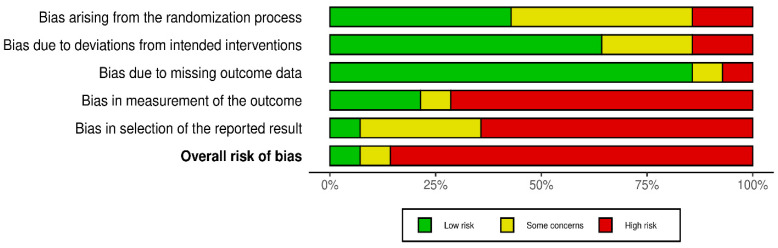
Summary of robvis for pain intensity outcomes.

**Figure 4:**
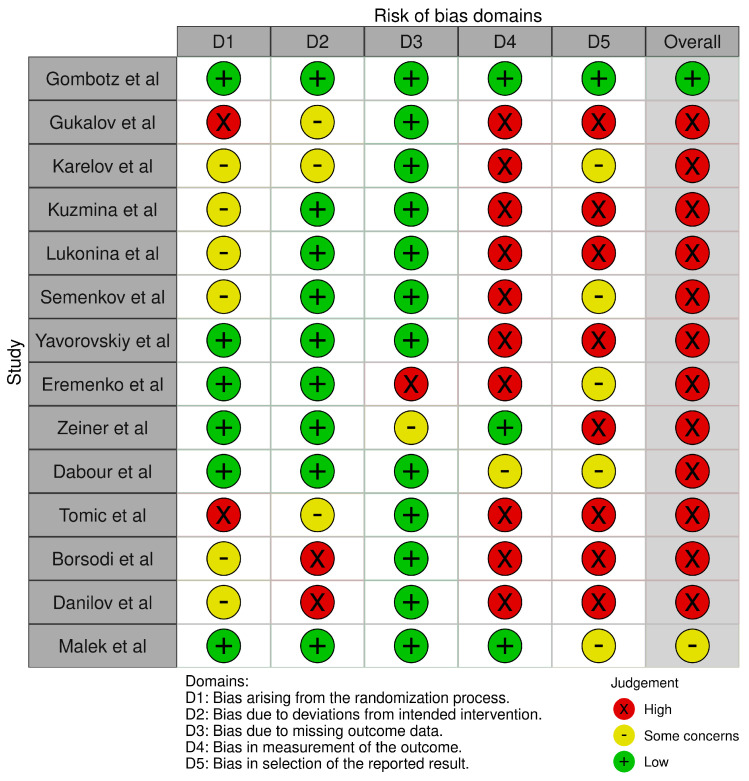
Robvis for opioid effect outcomes [27,28,29,30,31,32,33,34,35,36,37,38,39,40].

**Figure 5:**
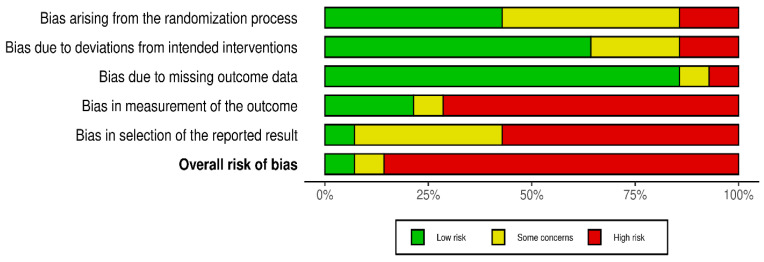
Robvis summary for opioid effect outcomes.

Robvis tool was used to visualize the bias measurements. This assessment was conducted per outcome [pain intensity (Figure 2 and Figure 3)] and opioid effect (Figure 4 and Figure 5).

### 3.4. Results of Individual Studies

The detailed PICO (population, intervention, comparator, outcome) characteristics and comprehensive results for all 14 included trials are provided in Supplementary Material S4. Briefly, the individual study findings regarding the efficacy of the fixed-dose combination (FDC) of Diclofenac and Orphenadrine are summarized below, grouped by surgical population.

The FDC was frequently evaluated in major visceral and cavity surgeries, generally demonstrating favorable analgesic and opioid-sparing outcomes. In thoracic surgery, Yavorovskiy et al. [35] reported that the FDC yielded significantly lower Visual Analog Scale (VAS) pain scores at rest and post-extubation up to 24 h compared to standard care, which corresponded with a three-fold reduction in total morphine consumption. Similarly, in cardiac and abdominal models, studies by Eremenko et al. [30], Karelov et al. [32], Danilov et al. [28], and Semenkov et al. [34] noted that the FDC provided an acceptable quality of postoperative analgesia accompanied by a significantly reduced need for rescue opioids such as tramadol or trimeperidine. While Danilov et al. [28] found comparable analgesia, they did not observe a significant reduction in opioid consumption specifically following thoracoscopic procedures. Eremenko et al. [30] reported that the FDC was effective in attenuating pain during movement and coughing, which is a critical component for early postoperative recovery.

In patients undergoing orthopedic procedures, the FDC consistently demonstrated opioid-sparing properties. Gombotz et al. [29] evaluated patients undergoing total hip arthroplasty and found a statistically significant reduction in 24 h Patient-Controlled Analgesia (PCA) morphine requirements for the FDC group compared to the placebo group (38.7 mg vs. 55.9 mg, *p* = 0.0004). Similarly, investigating major joint replacements, Gukalov et al. [38] evaluated hip arthroplasties and noted a trend toward lower pain scores at 24 h compared to morphine monotherapy, while Kuzmina et al. [39] reported significantly lower resting pain scores at 24 and 48 h following knee arthroplasty when compared to ketoprofen. Similarly, Zeiner et al. [36] investigated PCA hydromorphone requirements, comparing the FDC to both placebo and Diclofenac monotherapy, noting a trend toward reduced opioid demand in the first 24 h post-surgery when the FDC was utilized. Following arthroscopy, Málek et al. [33] reported encouraging analgesic effects for the FDC compared to placebo and piroxicam, with a significantly higher percentage of patients requiring no supplemental pethidine.

Several studies evaluated the FDC in general and oncological soft-tissue surgeries. Dabour et al. [27] demonstrated that the FDC significantly reduced resting pain scores at 12 and 24 h post-mastectomy compared to placebo, alongside a notable reduction in the need for rescue opioids; however, the Ketorolac+Orphenadrine combination was more effective. Borsodi et al. [31] evaluated patients undergoing hernia and breast surgeries, finding that the FDC safely and effectively reduced pain both at rest and during movement, leading to significantly lower cumulative tramadol consumption compared to tramadol monotherapy. In a broader oncological surgery population, Lukonina et al. [40] reported an acceptable level of analgesia, noting significant improvements in resting pain by the second postoperative day and a significantly shorter overall duration of postoperative opioid therapy prior to discharge.

Conversely, results in Orthognathic procedures were more equivocal. Tomić et al. [37] compared the FDC to ibuprofen following orthognathic surgeries (bimaxillary and bilateral sagittal split osteotomies). The authors found no significant differences in Numeric Rating Scale (NRS) pain scores or piritramide intake over the first two postoperative days; however, ibuprofen demonstrated statistically superior pain control on the third postoperative day, specifically within the bimaxillary surgery subgroup.

### 3.5. Results of the Synthesis

#### 3.5.1. Pain Intensity

A total of 13 studies were included in the synthesis to assess analgesia. Semenkov et al. [34] did not present raw mean VAS data and provided figures without SDs. Since we wouldn’t have the necessary data to compute the confidence intervals, we did not use any program to extract the data from the provided figure. Pain intensity was primarily assessed using the Visual Analog Scale (VAS) (11 studies [28,29,30,31,32,33,35,36,38,39,40]) and the Numerical Rating Scale (NRS) (2 studies [27,37]). Due to heterogeneity in statistical reporting, we stratified the synthesis into three distinct categories: VAS scores reported as means [28,29,30,31,32,33,36,40], VAS scores reported as medians [35,38,39,40], and NRS scores [27,37].

Before adding the data to the synthesis tables, specific adjustments to ensure accuracy and comparability were made:

- For Gombots et al. [29], where numerical data were not reported in the text, means and standard deviations (SD) were extracted from graphs using WebPlotDigitizer (version 4), a method aligned with Cochrane Handbook recommendations [24], because the authors omitted reporting the raw data themselves.
- For Danilov et al. [28], a likely typographical error in the comparator group’s standard deviation at the 6 h time point (reported as 2 rather than the expected range of 20) was assumed and adjusted for the analysis.
- For Lukonina et al. [40] reported both mean and median VAS values depending on the time point. We separated these data points to assess them in the respective synthesis tables.

Synthesis of Mean VAS Scores

**Figure 6:**
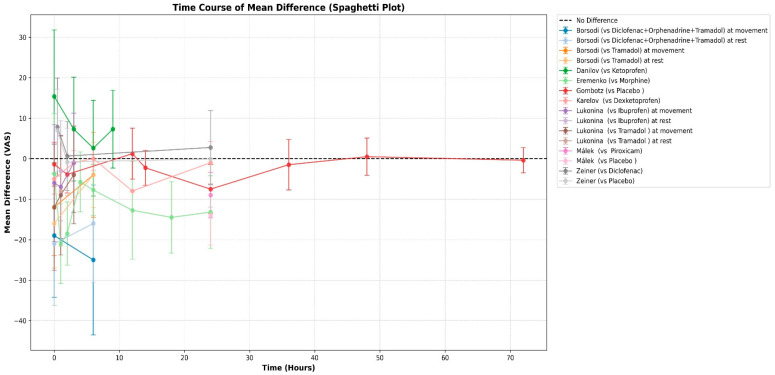
Spaghetti plot for the time course of the mean difference for all included studies. The *Y*-axis represents the mean difference in VAS scores between the treatment groups (Intervention minus Comparator), while the *X*-axis represents the specific postoperative time points. Unlike the subsequent forest plots, which evaluate efficacy at isolated 6 h and 24 h snapshots, this plot visualizes the overall dynamic trend of the FDC’s analgesic efficacy over the entire assessed postoperative period according to each study’s reported time points.

**Figure 7:**
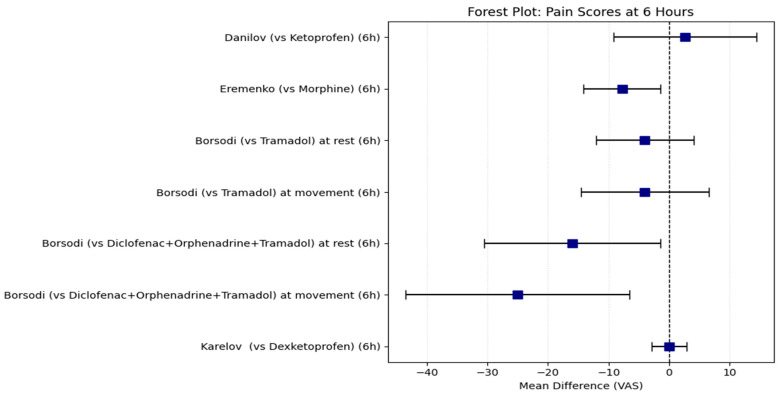
Forest plot of the MD of VAS scores at 6 h between treatment groups (Intervention-Comparator).

**Figure 8:**
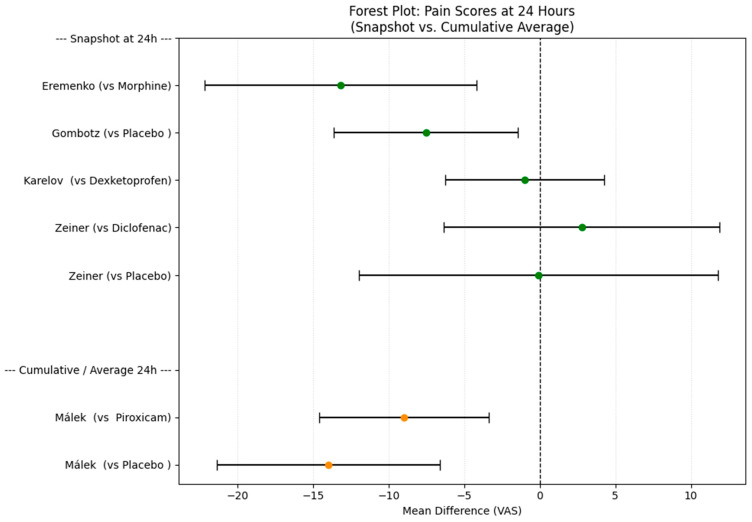
Forest plot of the mean difference in VAS scores at 24 h between treatment groups (Intervention–Comparator).

For studies reporting means, we first calculated the confidence intervals for the mean difference for all time points that each study reported, as described in the methods section (Table 5). A spaghetti plot to visualize the time course of the mean differences across all available time points was made to summarize everything (Figure 6). Additionally, forest plots (without summary diamonds) were generated for the most commonly reported time points across studies: 6 h (Figure 7) and 24 h (Figure 8).

Synthesis of Median VAS Scores

**Figure 9:**
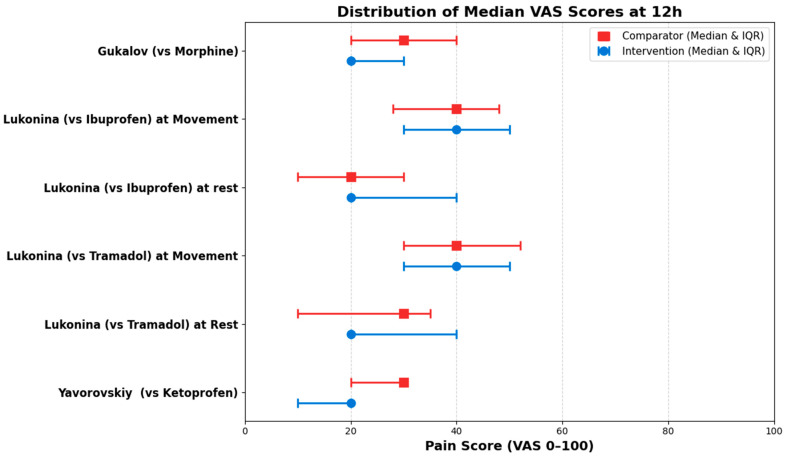
Distribution of the Median VAS raw data of intervention (blue) vs. comparator (red) at 12 h.

**Figure 10:**
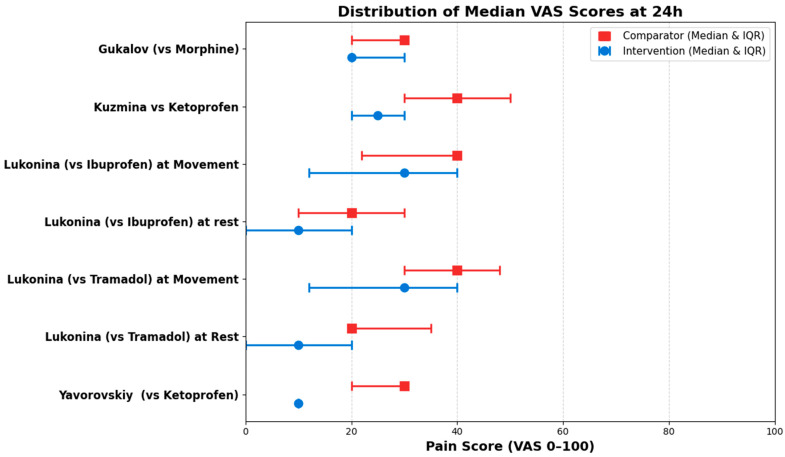
Distribution of the Median VAS raw data of intervention (blue) vs. comparator (red) at 24 h.

Due to the skewed nature of the data and the absence of individual patient data, accurate confidence intervals for median differences could not be derived. In accordance with Synthesis Without Meta-analysis (SWiM) reporting guidelines, the raw median values, their respective Interquartile Ranges (IQR), and the calculated median differences across all evaluated time points are detailed in Table 6. Furthermore, to visually represent the direction of the effect from this dataset, forest plots were constructed specifically for the 12 h (Figure 9) and 24 h (Figure 10) time points.

Synthesis of NRS Scores

**Figure 11:**
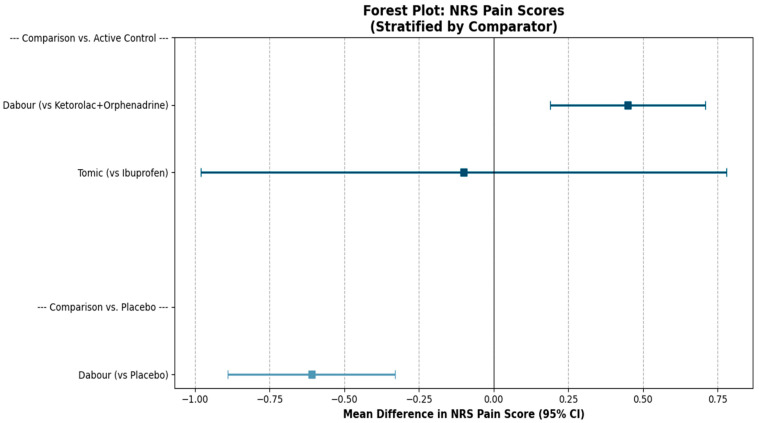
Forest plot of the mean difference in the NRS scores at 24 h between treatments (intervention–comparator).

To ensure methodological comparability during data synthesis, outcome data were harmonized by time window (Table 7). Since Tomic et al. [37]. reported NRS scores as an aggregate mean for the first postoperative day (derived from three daily measurements) rather than specific time points, we extracted the corresponding 0–24 h average NRS scores from Dabour et al. [27] to align the comparison. The pain scores were stratified by placebo or active control and are presented in a forest plot (Figure 11).

#### 3.5.2. Opioid Sparing Effect

Nine out of the fourteen included studies reported quantitative data on opioid consumption (mg consumed). To ensure comparability, all reported opioid dosages were converted into Morphine Milligram Equivalents (MME) using standard conversion calculators (EviQ). A specific adjustment was required for Trimeperidine (Promedol), a synthetic opioid reported in studies of Soviet origin. As this agent is not listed in standard Western MME calculators, we applied a conversion factor of 0.5 relative to morphine, based on available literature [53].

(A) Morphine Equivalent Milligrams Consumed

**Figure 12:**
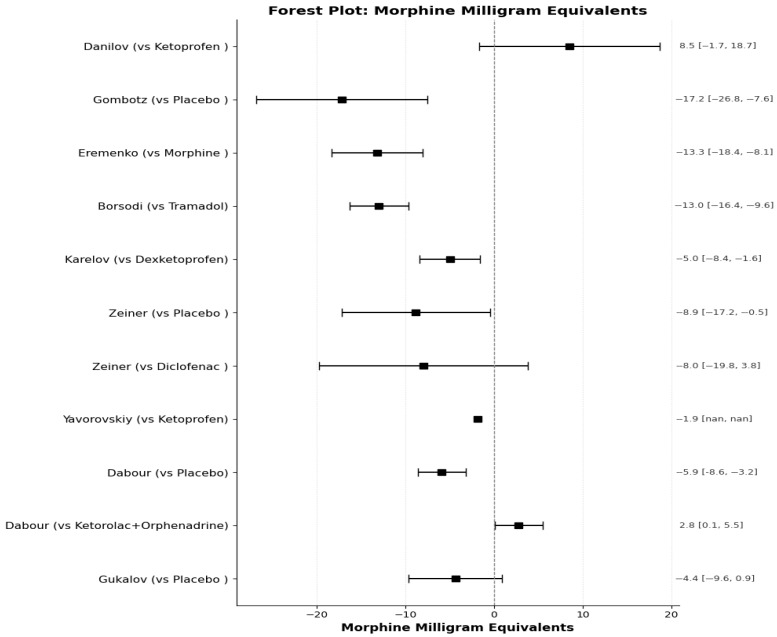
Forest plot of the mean difference in Morphine Milligram Equivalents consumption in 24 h of the intervention group compared to the control.

- The extracted data regarding Morphine Milligram Equivalents (MME) consumed over 24 h across the included studies, alongside the calculated mean differences, are synthesized in Table 8 and visually represented in the corresponding forest plot (Figure 12). However, several studies required specific extraction strategies to harmonize the data for this synthesis:Malek et al. [33], Lukonina et al. [40], Kuzmina et al. [39], and Semenkov et al. [34] did not report total milligrams consumed. Tomic et al. [37] reported total intake as an aggregate sum over three days without providing a mean per group or standard deviation, preventing inclusion in the pooled analysis; however, their reported results indicated similar intake levels between groups. The studies were excluded from the synthesis table of mg consumed.
- Dabour et al. presented consumption data only for the subset of patients requiring rescue analgesia. To enable an intention-to-treat comparison, we calculated the average for the whole treatment arm by combining the summary statistics of the two intervention subgroups (Diclofenac+Orphenadrine and Ketorolac+Orphenadrine) following Cochrane methods. Furthermore, a discrepancy was noted between the tabulated data for the placebo group (reporting 1.7–3.38 mg) and the text stating “significantly higher consumption.” Based on the data visualized in their Figure 2, we extrapolated that the placebo group consumed a total of 460 mg (at 5 mg per dose). We therefore calculated a corrected mean of 10.7 mg for the placebo arm (460 mg divided by 43 subjects) to accurately reflect the study’s findings.
- Borsodi et al. [27,31]: The study design included a split intervention group (FDC alone vs. FDC + Tramadol). To derive a representative value for the intervention arm, we merged the samples of the FDC and FDC+Tramadol groups. We calculated a weighted average of opioid consumption ([(19 × 0) + (11 × 61.5)]/30 = 22.55 mg Tramadol), converted this value to MME, and pooled the standard deviations using the sum of squares method.
- Gukalov et al. [38] presented opioid consumption as medians. As this was the only study in the quantitative synthesis reporting non-parametric data, we imputed the mean and standard deviation using the validated methods described by Luo et al. [54] and Wan et al. [55], respectively. A sensitivity analysis was conducted by excluding this study; the exclusion did not alter the overall direction of the effect.

(B) Use of Rescue Analgesia (% of patients)

**Figure 13:**
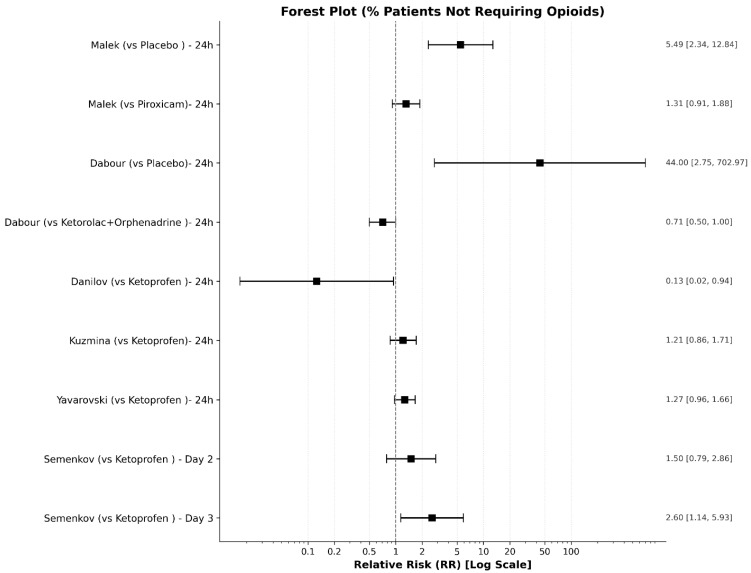
Forest plot for the risk ratio of the percent of patients not requiring opioids (intervention/comparator).

The data for the percentage of patients who did not require any rescue opioids across the relevant studies, along with the calculated relative risk (RR) ratios, are summarized in Table 9 and visualized in the accompanying forest plot (Figure 13). Six studies reported the percentage of patients who did not require any rescue opioids.

- Exclusions: Borsodi et al. [31] and Gukalov et al. [38] were excluded from this specific outcome as the comparator itself was an opioid. Karelov et al. [32], Tomic et al. [37], Lukonina et al. [40], Zeiner et al. [36], Eremenko et al. [30], and Gombots et al. [29] did not report binary data on rescue analgesia requirements.
- Correction: In Dabour et al. [27], 100% of patients in the placebo group required opioids, resulting in a zero value for “patients not requiring opioids.” To facilitate the calculation of the risk ratio (RR), we applied a correction by setting this value to 0.5, as done by other researchers [56].

#### 3.5.3. Safety

Regarding safety outcomes, the included studies exhibited significant heterogeneity in reporting standards, which precluded a uniform quantitative synthesis of all adverse events (AEs). 

Several studies compared FDC directly against ketoprofen. Yavorovskiy et al. [35] reported a significantly superior safety profile for the FDC group compared to ketoprofen. Specifically, the incidence of nausea was significantly lower in the FDC arm (5% vs. 40%, *p* = 0.008), as were drowsiness (25% vs. 70%, *p* = 0.004) and dry mouth (30% vs. 65%, *p* = 0.027). No significant differences were found regarding intestinal paresis or vomiting (vomiting incidence was 0 for the intervention group; thus, to calculate the RR, we used the value of 0.5 [57]). Furthermore, renal safety assessments (creatinine levels and average diuresis) confirmed the absence of kidney damage in both groups.

Similarly, Semenkov et al. [34] observed that nausea, drowsiness, and weakness were less common in the FDC group than in the ketoprofen group (*p* < 0.05). Biochemical markers, including average creatinine and CRP values, did not differ statistically, and Clavien-Dindo complications were comparable between arms.

Danilov et al. [28] found overall safety profiles to be comparable between FDC and ketoprofen regarding hemostatic parameters (platelet count, INR, fibrinogen, and D-dimer). However, they identified a potential benefit for FDC regarding renal safety: serum cystatin C levels were significantly lower in the FDC group compared to the control after 24 h of treatment (0.88 ± 0.34 mg/L vs. 1.19 ± 0.44 mg/L; *p* = 0.031).

In a comparison with piroxicam, Málek et al. [33] reported that FDC resulted in fewer total adverse events than both piroxicam and placebo (*p* < 0.05), though the specific types of events were not detailed.

**Figure 14:**
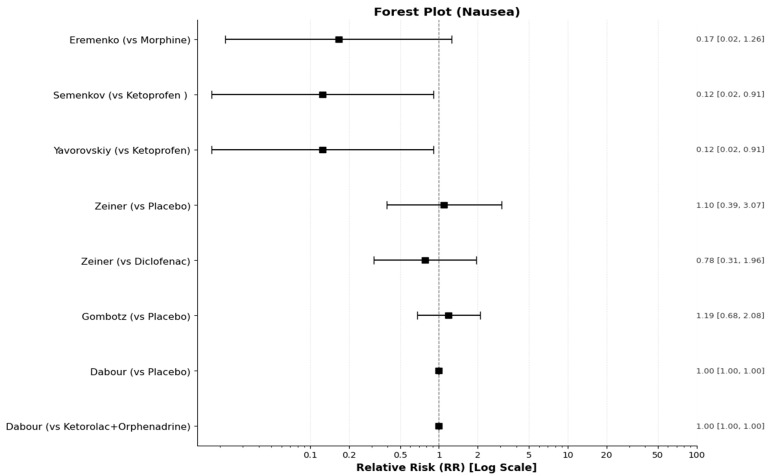
Forest plot for the RR of nausea incidence between treatments (intervention/comparator).

**Figure 15:**
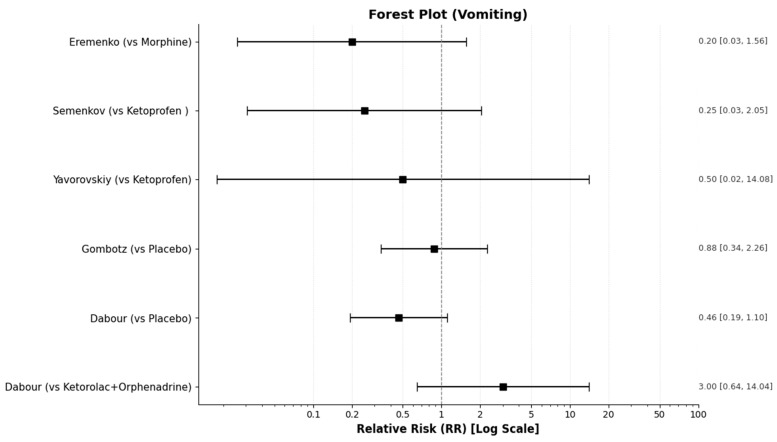
Forest plot for the RR of vomiting incidence between treatments (intervention/comparator).

Dabour et al. [27] focused on postoperative nausea and vomiting (PONV). They reported that the placebo group had a significantly higher incidence of high PONV scores compared to active treatment groups. A score of 0 for vomiting was achieved by 95.4% of the ketorolac+Orphenadrine group and 86% of the Diclofenac+Orphenadrine group, compared to only 69.8% of the placebo group (*p* = 0.016). Consequently, the placebo group required significantly more antiemetic rescue treatment. Since all the other studies reported only the incidence of vomiting and nausea and not severity by score, we pooled these cases in order to compare them in the synthesis table (Table 10) and the corresponding forest plots (Figure 14 and Figure 15). 

Eremenko et al. [30] noted that nausea and vomiting were more frequent in the morphine group compared to the FDC group. The authors reported that the incidence of other adverse events, like drowsiness, was more common in the morphine monotherapy group, but the full table of adverse events was not retrieved in their paper [30]. Conversely, Gukalov et al. found the incidence of AEs (including nausea, vomiting, and itching) to be comparable between FDC and morphine (15% in both groups) [38]. However, they presented these AEs pooled; thus, we could not extract the RR for each AE separately.

Gombotz et al. [29] and Zeiner et al. [36] found no significant differences between FDC and placebo/control groups. Gombotz et al. [29] reported comparable AE incidence with no significant deviations in vital signs or laboratory measurements [29]. Zeiner et al. [36] reported no severe adverse events, with comparable nausea rates across FDC (26.1%), Placebo (23.8%), and Diclofenac (33.3%) groups.

Borsodi et al. reported that physician-rated tolerability was “very good” or “good” across all three study groups. While three AEs were recorded (two nausea, one vomiting), the authors did not specify the incidence of these by group, precluding inclusion in the synthesis [31]. Karelov et al. [32] did not evaluate safety outcomes. Finally, Tomic et al. [37], Lukonina et al. [40], Kuzmina et al. [39] reported that no adverse events occurred in their respective studies.

The only adverse events consistently reported across multiple studies were nausea and vomiting. The detailed synthesis of these events is presented in the table below (Table 10), while the relative risks (RR) for the incidence of nausea and vomiting between treatments are visualized in Figure 14 and Figure 15, respectively:

### 3.6. Certainty of Evidence

The certainty of evidence for the outcomes of this review was assessed using the GRADE framework and is summarized in Table 11. We included seven of the eight outcomes reported in the summary table: VAS at 6 h and 24 h, NRS scores at 24 h, milligrams of morphine equivalents consumed, opioid sparing effect, and nausea and vomiting, since for VAS median values, we did not calculate the difference or its effect. Overall, the quality of evidence was rated as very low for all outcomes. The primary reason for downgrading was the risk of bias across studies, where almost all of them were judged as high. The only exception was the certainty of evidence for the pain (VAS) at 24 h, which was downgraded by only one level (−1) rather than two, since approximately 50% of the participant data for this outcome was contributed by studies with low-to-moderate risk of bias (Gombotz et al. [29], Malek et al. [33]). All the other outcomes included more than 50% of subjects from studies rated with high bias. For the inconsistency, since this was a SWIM and not a meta-analysis, we did not use any statistical test for heterogeneity. This factor was assessed visually, taking into consideration the overlapping of CI from individual studies and the direction of effect. The CIs and the direction of effect were not clear for the outcomes and were downgraded, respectively. Lastly, for imprecision, all outcomes demonstrated wide confidence intervals that crossed the line of no effect or included both appreciable benefit and harm and were downgraded as such.

## 4. Discussion

The objective of this systematic review was to evaluate the efficacy and safety profile of fixed-dose combinations (FDC) of Diclofenac and Orphenadrine for postoperative pain management, utilizing a Synthesis Without Meta-analysis (SWiM) approach [26] due to high clinical and methodological heterogeneity. The synthesis suggests that the FDC may improve analgesic efficacy and may exert opioid-sparing effects when compared to active comparators or placebo, although the evidence is very uncertain. Due to high bias and heterogeneity of the included studies, the quality of evidence is very low, and the results should be interpreted with caution.

While the synergistic benefits of combining NSAIDs with muscle relaxants have been broadly reviewed [22,58], to our knowledge, no dedicated systematic review has specifically synthesized the evidence for the fixed-dose combination of Diclofenac and Orphenadrine. This review addresses this gap by providing the first systematic evaluation of its efficacy and safety profile in postoperative pain. Consistent with this rationale, the multimodal mechanism of action, combining an NSAID (Diclofenac) with a muscle relaxant (Orphenadrine), appeared effective in reducing patient-reported pain scores while lowering the requirement for rescue opioid consumption.

The findings regarding pain relief must be interpreted in the context of concomitant opioid consumption, reflecting the clinical interdependence of these outcomes. As emphasized in the IMMPACT guidelines, the validity of analgesic trials depends on the integrated assessment of pain intensity and rescue medication use since assessing pain scores in isolation may yield misleading conclusions [59]. The observed reduction in pain was frequently achieved alongside a trend towards lower opioid consumption, supporting the benefit of the combination therapy in the setting of acute surgical pain.

Furthermore, the data collected on adverse events reinforces the clinical benefit derived from reduced opioid use. In studies where opioid consumption was substantially decreased, a correspondingly lower incidence of common, dose-dependent AEs (such as nausea and vomiting) was noted. Previous association on this has already been established by other authors [60]. However, the confidence in these efficacy and safety estimates remains severely restricted by the methodological limitations discussed below.

The certainty of the evidence regarding the efficacy and safety of the FDC for postoperative pain is severely limited. Across all evaluated outcomes (e.g., pain, opioid use, adverse events), the certainty was formally graded as “Very Low” using the GRADE methodology. This critical limitation is primarily due to endemic methodological deficiencies identified in the included studies. Of the 14 included randomized controlled trials (RCTs) encompassing 981 participants, only one study [29] was classified as having a low overall risk of bias. The remaining 13 studies were judged to have a high overall risk of bias or some concerns. The risk of bias was predominantly driven by issues in outcome measurement and the selection of reported results. The critical issues identified were the pervasive absence of Statistical Analysis Plans (SAPs) or public protocols across studies, and the lack of blinding in most of the studies, leading to high bias scores. The lack of transparency raises the possibility of selective reporting, wherein favorable differences at specific time points were highlighted while non-significant results were omitted. Additionally, the variability in study characteristics, intervention doses, comparators, surgical operation types, and perioperative administered medications (e.g., anesthetics) resulted in high heterogeneity, necessitating the SWiM approach. Furthermore, the majority of the point estimates provided by the majority of the included studies possessed wide confidence intervals, indicating an insufficient sample size or wide random error around the true effect. Furthermore, the internal validity of the included trials is threatened by the pervasive issue of perioperative polypharmacy, where the concomitant use of multiple anesthetic and analgesic agents confounds the isolation of the FDC’s specific effects. Thus, claims of it acting as a panacea for postoperative pain are unsupported by the current high-bias evidence. Lastly, the mitigation of dynamic pain is a primary objective in postoperative care to facilitate early mobilization. As highlighted by the European Medicines Agency (EMA) [61], depending on the clinical situation, pain measurements should be performed not only at rest but also on movement or after applying an appropriate stimulus; pain on movement is very important for function, whereas pain at rest correlates more with comfort. Despite this regulatory standard, the reporting of this outcome was highly inconsistent across the literature. The vast majority of the included studies only reported generic or resting pain scores, with some explicitly noting the lack of pain assessment during joint movement as a major study limitation (e.g., Zeiner et al. [36]). Because few studies differentiated between resting pain and pain upon movement, the overall impact of the FDC specifically on dynamic pain cannot be reliably synthesized.

We would like to acknowledge many limitations of our study. The primary limitation arising from the review process is the limited interpretability inherent in the SWiM method. Since meta-analysis of effect estimates was deemed inappropriate due to heterogeneity and poor reporting, results were synthesized descriptively using tools based on the direction of effect. While transparent, this approach provides more limited information for decision-making. Furthermore, during data synthesis, the time points of pain measurement were not consistent between the included studies. We selected the 6 h and the 24 h since the majority of these reported data are in these time points, but this may obscure fluctuations in analgesic efficacy occurring in other time points. Furthermore, we could not reliably determine if the FDC is more effective in one specific surgical model over another. The limited number of studies per surgical category, combined with the extreme clinical heterogeneity of the control groups, which utilized drastically different active comparators and varying rescue opioid protocols (e.g., morphine, tramadol, trimeperidine), introduces severe confounding. Another limitation of this study was that most of the included studies were in non-English languages (e.g., Russian, Czech); thus, the extracted data could contain translation inaccuracies. Furthermore, data quality issues and reporting errors in the included studies necessitated significant reviewer interventions during data extraction. In the study by Dabour et al. [27], a critical discrepancy was identified where the tabulated opioid consumption for the placebo group (1.7 mg) contradicted both the study’s textual conclusions and graphical representations (indicating ~10.7 mg). To correct this reporting error, control group values were extrapolated directly from the figures, introducing a potential for investigator estimation error. For the study by Gukalov et al. [38], continuous outcomes reported as medians were imputed to means and standard deviations using validated algorithms to facilitate synthesis. While excluding this study did not alter the overall directional trends, this transformation assumes a normal distribution that may not fully reflect the skewness of the original non-parametric data. Another methodological limitation of our review process is that the study protocol was not prospectively registered prior to study initiation. Although we strictly adhered to an internal procedure and retrospectively registered our methodology on the Open Science Framework (OSF) to maximize transparency, the lack of prospective registration limits the ability to independently verify that no data-driven deviations occurred during the review process. Furthermore, our search strategy carries methodological limitations. The search was restricted to generic drug names without the use of controlled vocabulary (e.g., MeSH), brand names (e.g., Neodolpasse), or spelling variants, which could theoretically omit relevant records. Additionally, the search strategy was not formally peer-reviewed by an independent information specialist using the PRESS (Peer Review of Electronic Search Strategies) framework. Furthermore, for Gombotz et al. [29], numerical data were not reported in the text and had to be extracted from graphs using digital software (WebPlotDigitizer). While this aligns with Cochrane guidance, relying on pixel-based extraction from published images introduces potential imprecision in measurement.

Finally, because the extreme heterogeneity of the data precluded a formal meta-analysis, we were unable to quantitatively assess publication bias or small-study effects using funnel plots or statistical tests for asymmetry. Such quantitative methods require a pooled statistical synthesis of at least 10 studies to achieve adequate power. To minimize the risk of non-reporting biases, we conducted a comprehensive search of gray literature and clinical trial registries. This search successfully identified unpublished records, including two conference abstracts (Grecu 2008 [62], Ologoiu 2008 [63]) and one incomplete trial registry (APOPKA/NCT02456116 [64]). In accordance with our inclusion criteria, these records were excluded from the formal synthesis because the lack of full-text data prevents the rigorous assessment of methodological quality via the RoB2 tool. However, a qualitative review of their contents revealed that both abstracts reported positive analgesic and opioid-sparing effects for the fixed-dose combination, while the registry contained no posted results. Consequently, the exclusion of these unpublished reports does not suppress negative findings. Rather, the existence of these small, unpublished positive trials strongly reinforces our concern regarding small-study effects, where smaller, methodologically limited trials report predominantly positive effects. This qualitative assessment of publication bias further justifies our highly cautious interpretation of the findings and our decision to downgrade the overall certainty of the evidence to ‘Very Low’ via the GRADE framework.

An important contextual observation from this systematic review is the geographical distribution of the included trials, which predominantly originate from specific regions such as Russia, Austria, Hungary, the Czech Republic, and Egypt. This clustering could be explained by the regulatory and commercial landscape of this specific fixed-dose combination. The primary intravenous formulation of Diclofenac and Orphenadrine (Neodolpasse) is manufactured in Austria and is widely registered and utilized within these specific European and neighboring markets; however, the underlying reasons for this specific regional research interest cannot be definitively determined from the synthesized clinical data. Consequently, this geographical limitation must be considered, as the findings may have limited generalizability to healthcare systems with different standard analgesic practices or where this specific combination is unavailable.

Given that the certainty of the evidence is Very Low, specific clinical recommendations for practice cannot be made. While the direction of effect suggests that fixed-dose Diclofenac and Orphenadrine may be a beneficial component of a multimodal analgesic strategy for postoperative pain, the very low certainty of the evidence precludes drawing firm conclusions. Clinicians and policy makers may consider the consistent direction of effect demonstrating a potential opioid-sparing benefit achieved with this FDC, as reducing narcotic consumption is a key objective for both safety and public health. Decision-makers must weigh the potential benefits shown by the observed directional trends against the high uncertainty in the estimated effect magnitude.

The critical limitations identified in this systematic review mandate clear recommendations for future research. The RCTs must be prospectively registered, and the Statistical Analysis Plans (SAPs) should be publicly accessible before study completion. New studies must address other structural biases, specifically providing detailed reports on the blinding of outcome assessors and minimizing participant attrition, to ensure the resulting evidence is at a low risk of bias. Additionally, the protocols must standardize intraoperative anesthesia to reduce the noise observed. Lastly, future trials should use standardized outcome measures and time points as explained in pain guidelines [61,65] to reduce clinical and methodological inconsistency. This standardization would allow for subsequent statistical synthesis (meta-analysis) and provide more precise estimates of effect, thereby addressing the issue of imprecision in the current evidence base.

## 5. Conclusions

The fixed-dose combination (FDC) of Diclofenac and Orphenadrine represents a rational, mechanism-based approach to postoperative analgesia. The synthesis demonstrated that the FDC may improve pain and may reduce opioid consumption compared to active comparators and placebo. This opioid-sparing effect is clinically relevant, as it could correlate with a reduced incidence of dose-dependent adverse events such as nausea and vomiting, thereby supporting the utility of this combination as a valuable component of multimodal analgesic regimens intended to minimize the perioperative opioid burden.

However, the current body of evidence is compromised by very low certainty, driven by reporting biases, confounding medications used in anesthesia, and methodological flaws. Specifically, because all included studies were judged to be at a high risk of bias, the apparent efficacy findings must be interpreted with caution. The poor overall study quality inherently limits the ability to draw firm clinical conclusions regarding the true effect of the combination therapy. Therefore, clinical practice should remain cognizant of the limitations in the supporting data until high-quality, pre-registered confirmatory trials are available. Future research requires low-bias, pre-registered RCTs to confirm these findings with greater precision.

## Figures and Tables

**Table 1:** PICO-based inclusion criteria.

PICO Component	Criteria for Inclusion
P (Population)	Adult patients (>18 years) undergoing various surgeries requiring postoperative pain relief.
I (Intervention)	Fixed combination of Diclofenac and Orphenadrine.
C (Comparator)	Placebo, Diclofenac alone, other analgesics, including opioids or NSAIDs.
O (Outcome)	Postoperative pain intensity (VAS/NRS scores), opioid sparing effect, and incidence of adverse effects.

**Table 2:** Excluded studies from the systematic review.

Study	Reason for Exclusion
Sedláčková, E., & Černý, D., 2020 [41]	This study presented a case series
Sorokina et al. 2022 [42]	This study was a retrospective study
Visic et al., 2021 [43]	This study was a retrospective study
Amelin et al., 2021 [44]	This study presented 3 case reports with acute pain
Amelin et al., 2022 [45]	This was an observational study (NEODOLEX) evaluating acute non-specific pain
Zyryanov et al., 2021 [46]	This study presented 4 case reports with dorsalgia
Shirokov et al., 2021 [47]	This study evaluated patients with acute spondylogenic pain syndromes and not post-operative pain
Schaffler et al., 2005 [48]	This study evaluated the pain relief in capsaicin-induced pain models
Ghafoor et al., 2019 [49]	This was a cross-sectional study
Veerabhadrappa et al., 2021 [50]	This study evaluated myogenous masticatory pain and not post-operative pain
Uitz et al., 1998 [51]	This study evaluated patients with osteoarthritis-related chronic pain
Abuzarova et al., 2022 [52]	This study evaluated patients after radiotherapy, but it had an ineligible comparator (Diclofenac and Orphenadrine combination was used in both groups with different doses, thus making it a dose-controlled study, not an active-controlled one)

**Table 3:** Characteristics of included studies.

Study	Pooled Patients (*N* = 981)	Female vs. Men	Operation Type
Dabour, 2023 [27]	129 women (43 per group)	All women (0% men)	Mastectomy
Danilov, 2024 [28]	47 patients (25 study, 22 control)	Both sexes (comparable groups)	Planned Thoracoscopic operations
Eremenko, 2022 [30]	40 patients (20 per group)	Sex distribution comparable	Cardiac surgery
Gukalov, 2023 [38]	65 patients (39 study, 26 control)	Sex distribution comparable	Hip arthroplasty
Karelov, 2023 [32]	48 patients (24 per group)	24 men, 24 women (Abdominal: 15 men, 9 women; Spinal: 9 men, 15 women)	Abdominal surgeries OR Vertebral surgeries
Kuzmina, 2023 [39]	40 patients (20 per group)	7 men, 33 women (5/15 main, 2/18 comparison)	Knee arthroplasty
Lukonina, 2024 [40]	52 patients (15 K/Tramadol, 18 D/D+O, 19 I/Ibuprofen)	23 men (44.2%), 29 women (55.8%)	Spinal surgery
Málek, 2004 [33]	119 patients (44 Piroxicam, 35 Neodolpasse, 40 Control/Placebo)	Not specified by count	Knee arthroscopy
Semenkov, 2024 [34]	40 cancer patients (20 Neodolpasse, 20 Ketoprofen)	Not specified by count	Abdominal surgery
Tomic, 2022 [37]	109 patients enrolled (53 Diclofenac+Orphenadrine, 56 Ibuprofen)	Not specified by count	Orthognathic surgery
Yavorovskiy, 2023 [35]	40 patients (20 Neodolpasse, 20 Ketoprofen)	Not specified by count	Thoracic surgery
Zeiner, 2023 [36]	65 patients completed (72 initially randomized) (21 Placebo, 21 Diclofenac, 23 Diclofenac+Orphenadrine)	27.7% female, 72.3% male (Overall *N* = 65)	Elective cruciate ligament surgery (arthroscopic reconstruction)
Gombotz, 2010 [29]	120 patients (60 Verum/D+O, 60 Placebo)	Not specified by count	Hip Arthroplasty
Borsodi, 2008 [31]	60 patients (19 D+O only, 30 Tramadol only, 11 D+O + Tramadol)	42 female, 18 male	Minor and moderate surgeries (open hernia surgery, breast surgery, hydrocele testis)

Abbreviations: N: number of patients; D+O: Diclofenac and Orphenadrine; vs.: versus.

**Table 4:** Main efficacy measurements in the included studies.

	Dabour et al. [27]	Danilov et al. [28]	Eremenko et al. [30]	Gukalov et al. [38]	Karelov et al. [32]	Kuzmina et al. [39]	Lukonina et al. [40]	Málek et al. [33]	Semenkov et al. [34]	Tomic et al. [37]	Yavorovskiy et al. [35]	Zeiner et al. [36]	Gombotz et al. [29]	Borsodi et al. [31]
VAS		√	√	√	√	√	√	√	√		√	√	√	√
NRS	√									√				
Opioid effect	√	√	√	√	√	√	√	√	√	√	√	√	√	√
VRS													√	
Non-opioid rescue consumption					√					√				
PSS	√						√							√
SSS	√						√							√
Lab measurements		√					√		√		√	√	√	
PRS														√
MILC			√								√			
WBF						√								
HD							√		√	√				
RSS	√													
Safety	√	√	√	√		√	√	√	√	√	√	√	√	√

Abbreviations: PSS: patient satisfaction score; SSS: surgeon satisfaction score; MILC: maximum inspiratory capacity of the lungs, WBF: weight-bearing fraction, PRS: physician-rated safety; NRS: numeric rate scale; VAS: Visual Analog Scale; VRS: Verbal Rating Scale; RSS: Ramsey sedation score; HD: hospitalization days.

**Table 5:** Synthesis table of studies presenting raw VAS scores as means.

Study	Comparator	Time After Surgery	Mean VAS Scores in mm (±SD)		Mean Difference (MD)	Upper, Lower 95% CI
			Intervention	Comparator		
Danilov et al., 2024 [28] (*n* = 47)	Ketoprofen (*n* = 22)	0 h	58.7 ± 29.5	43.3 ± 26.4	15.4 ± 8.15	[−1.01, 31.81]
		3 h	44 ± 24.1	36.7 ± 19.5	7.3 ± 6.37	[−5.53, 20.13]
		6 h	37.3 ± 20.2	34.7 ± 20	2.6 ± 5.87	[−9.22, 14.42]
		9 h	35.3 ± 16.0	28.0 ± 16.6	7.3 ± 4.77	[−2.31, 16.91]
Gombotz et al., 2010 [29] 2004-002724-17 (*n* = 120)	Placebo (*n* = 60)	0 h (Baseline/Before 1st Infusion)	2.96 ± 15.55	4.335 ± 14.465	−1.37 ± 2.74	[−6.8, 4.05]
		~1.25 after 1st infusion	3.685 ± 9.755	7.585 ± 15.115	−3.9 ± 2.32	[−8.49, 0.69]
		~12 h before 2nd infusion	12.415 ± 19.095	11.185 ± 15.575	1.23 ± 3.18	[−5.07, 7.53]
		~13.5 h after 2nd infusion	4.26 ± 12.09	6.52 ± 11.75	−2.26 ± 2.18	[−6.58, 2.06]
		Morning Day 2	8.74 ± 13.89	16.275 ± 19.435	−7.535 ± 3.08	[−13.63, −1.44]
		Afternoon Day 2	10.535 ± 16.915	12.025 ± 17.565	−1.49 ± 3.15	[−7.73, 4.75]
		Morning Day 3	8.85 ± 12.32	8.355 ± 13.045	0.49 ± 2.32	[−4.1, 5.09]
		Afternoon Day 3	3.99 ± 6.85	4.375 ± 10.145	−0.385 ± 1.58	[−3.51, 2.74]
Eremenko et al., 2022 [30] NCT05322603 (*n* = 40)	Morphine monotherapy (*n* = 20)	0 h	41 ± 24.27	44.75 ± 22.09	−3.75 ± 7.33	[−18.59, 11.09]
		1 h	19 ± 14.74	40 ± 15.89	−21 ± 4.85	[−30.82, −11.18]
		2 h	17 ± 11.16	35.5 ± 13.16	−18.5 ± 3.86	[−26.31, −10.69]
		4 h	16.75 ± 9.9	22.5 ± 13.02	−5.75 ± 3.66	[−13.16, 1.66]
		6 h	16 ± 8.3	23.75 ± 11.34	−7.75 ± 3.14	[−14.11, −1.39]
		12 h	20.75 ± 15.99	33.5 ± 21.34	−12.75 ± 5.96	[−24.82, −0.68]
		18 h	15.75 ± 10.03	30.25 ± 16.73	−14.5 ± 4.36	[−23.33, −5.67]
		24 h	14.55 ± 9.5	27.75 ± 17.43	−13.2 ± 4.44	[−22.19, −4.21]
Borsodi et al., 2008 [31] (*n* = 60)	Tramadol (*n* = 30)	0 h rest	32 ± 17	48 ± 21	−16 ± 5.47	[−27, −5]
		0 h movement	46 ± 19	58 ± 22	−12 ± 5.93	[−23.93, −0.07]
		6 h rest	7 ± 11	11 ± 17	−4 ± 4	[−12.05, 4.05]
		6 h movement	16 ± 17	20 ± 19	−4 ± 5.22	[−14.5, 6.5]
	Diclofenac+Orphenadrine + Tramadol (*n* = 11)	0 h rest	32 ± 17	53 ± 21	−21 ± 7.44	[−36.24, −5.76]
		0 h movement	46 ± 19	65 ± 20	−19 ± 7.44	[−34.24, −3.76]
		6 h rest	7 ± 11	23 ± 22	−16 ± 7.1	[−30.54, −1.46]
		6 h movement	6 ± 17	31 ± 27	−25 ± 9.03	[−43.5, −6.5]
Karelov et al., 2023 [32] (*n* = 48)	Dexketoprofen (*n* = 24)	0 h (Awakening) Note: FDC was received during surgery, while ketoprofen was administered after awakening	30 ± 6	35 ± 7	−5 ± 1.88	[−8.78, −1.22]
		3 h	39 ± 4	40 ± 6	−1 ± 1.47	[−3.96, 1.96]
		6 h	53 ± 5	53 ± 5	0 ± 1.44	[−2.9, 2.9]
		12 h	41	49	−8	-
		24 h	38 ± 10	39 ± 8	−1 ± 2.61	[−6.25, 4.25]
Zeiner, 2023 [36] NCT03493490 (*n* = 72)	Placebo (*n* = 21)	30 min	57.35 ± 12.97	50.33 ± 19.45	7.02 ± 5.03	[−3.13, 17.17]
		2 h	29.70 ± 13.42	30.52 ± 13.88	−0.82 ± 4.12	[−9.13, 7.49]
		24 h	28.48 ± 16.48	28.57 ± 21.92	−0.09 ± 5.89	[−11.98, 11.8]
	Diclofenac (*n* = 21)	30 min	57.35 ± 12.97	49.52 ± 24.59	7.83 ± 6.01	[−4.3, 19.96]
		2 h	29.70 ± 13.42	29.05 ± 14.46	0.65 ± 4.22	[−7.87, 9.17]
		24 h	28.48 ± 16.48	25.71 ± 13.54	2.77 ± 4.53	[−6.37, 11.91]
Lukonina et al., 2024 [40] (*n* = 52)	Ibuprofen (*n* = 19)	Transfer to ICU (movement) (IMP was given 30 min before the end of surgery)	34 ± 19	40 ± 24	−6 ± 7.1	[−20.41, 8.41]
		1 h rest	21 ± 19	24 ± 18	−3 ± 6.09	[−15.36, 9.36]
		1 h (movement)	33 ± 19	40 ± 19	−7 ± 6.25	[−19.69, 5.69]
		3 h (movement)	37 ± 14	38 ± 22	−1 ± 6.03	[−13.24, 11.24]
	Tramadol monotherapy (*n* = 15)	Transfer to ICU (movement) (IMP was given 30 min before the end of surgery)	34 ± 19	46 ± 24	−12 ± 7.65	[−27.6, 3.6]
		1 h rest	21 ± 19	29 ± 19	−8 ± 6.64	[−21.54, 5.54]
		1 h (movement)	33 ± 19	42 ± 22	−9 ± 7.23	[−23.75, 5.75]
		3 h (movement)	37 ± 14	41 ± 19	−4 ± 5.91	[−16.05, 8.05]
Málek et al., 2004 [33] (*n* = 119)	Placebo (*n* = 40)	Average scores during 24 h	15 ± 15	29 ± 17	−14 ± 3.7	[−21.37, −6.63]
	Piroxicam (*n* = 44)	Average scores during 24 h	15 ± 15	24 ± 18	−9 ± 2.81	[−14.6, −3.4]

Abbreviations: VAS: Visual Analog Scale; SD: standard deviation; MD: mean difference; CI: confidence interval. Note: Colored cells indicate the direction of effect. Green indicates an effect estimate favoring the fixed-dose combination (Diclofenac/Orphenadrine), while orange indicates an effect estimate favoring the comparator or representing no significant difference.

**Table 6:** Synthesis table of studies presenting raw VAS scores as medians.

Study	Comparator	Time After Surgery	Median VAS Scores in mm (Q1–Q3)		Median Difference
			Intervention Group	Comparator	
Lukonina et al., 2024 [40] (*n* = 52)	Ibuprofen (*n* = 19)	0 h (rest)	5 (0–30)	10 (0–30)	−5
		0 h (movement)	30 (10–48)	30 (20–40)	0
		Transfer to the ICU rest	20 (0–38)	20 (5–40)	0
		3 h rest	20 (10–30)	20 (8–40)	0
		12 h rest	20 (20–40)	20 (10–30)	0
		12 h movement	40 (30–50)	40 (28–48)	0
		24 h rest	10 (0–20)	20 (10–30)	−10
		24 h movement	30 (12–40)	40 (22–40)	−10
	Tramadol (*n* = 15)	0 h (rest)	5 (0–30)	15 (5–30)	−10
		0 h (movement)	30 (10–48)	30 (18–42)	0
		Transfer to the ICU rest	20 (0–38)	30 (18–40)	−10
		3 h rest	20 (10–30)	20 (20–40)	0
		12 h rest	20 (20–40)	30 (10–35)	−10
		12 h movement	40 (30–50)	40 (30–52)	0
		24 h rest	10 (0–20)	20 (20–35)	−10 (*p* = 0.025)
		24 h movement	30 (12–40)	40 (30–48)	−10
Kuzmina et al. 2023 [39] (*n* = 40)	Ketoprofen (*n* = 20)	6 h	No significant difference was reported by the authors between groups.		-
		12 h	Data measured but not reported. The authors note a more favorable trend for FDC in later periods.		-
		24 h	25 (20–30)	40 (30–50)	−15 (*p* = 0.006)
		48 h	20 (20–30)	30 (28–40)	−10 (*p* = 0.021)
Gukalov et al., 2023 [38] (*n* = 65)	Morphine only (*n* = 26)	2 h	50 (40–60)	50 (40–60)	0 (ns, *p* = 0.813)
		12 h	20 (20–30)	30 (20–40)	−10 (ns, *p* = 0.071)
		24 h	20 (20–30)	30 (20–30)	−10 (*p* = 0.05)
Yavorovskiy et al., 2023 [35] (*n* = 40)	Ketoprofen (*n* = 20)	0 h	30 (20–40)	40 (30–60)	−10 (*p* = 0.096)
		1 h	20 (20–30)	40 (30–40)	−20 (*p* < 0.001)
		2 h	30 (20–30)	35 (30–40)	−5 (*p* = 0.001)
		4 h	30 (20–30)	30 (30–40)	0 ns, *p* = 0.096
		6 h	20 (20–30)	30 (30–40	−10 (*p* < 0.001)
		8 h	20 (10–30)	30 (30–40)	−10 (*p* < 0.001)
		10 h	20 (20–20)	30 (20–40)	−10 (*p* < 0.001)
		12 h	20 (10–20)	30 (20–30)	−10 (*p* < 0.001)
		18 h	15 (10–20)	20 (20–40)	−5 (*p* = 0.006)
		24 h	10 (10–10)	30 (20–30)	−20 (*p* < 0.001)

Abbreviations: VAS: Visual Analog Scale; Q1–Q3: interquartile range (25th–75th percentile); FDC: fixed-dose combination; ns: not significant; *p*: *p*-value. Note: Colored cells indicate the direction of effect. Green indicates an effect estimate favoring the fixed-dose combination (Diclofenac/Orphenadrine), while orange indicates an effect estimate favoring the comparator or representing no significant difference.

**Table 7:** Synthesis table of studies presenting raw NRS scores as means.

Study	Comparator	Time After Surgery	NRS Mean Scores		Mean Difference (95% CI Upper, 95% CI Lower)
			Intervention Group	Comparator	
Tomic, 2022 [37] (*n* = 109)	Ibuprofen (*n* = 56)	Day 0 PO (Average of 3 measurements)	2.58 (95% CI 1.94–3.23)	2.39 (95% CI 1.84–2.94)	0.19 [−0.65, 1.03]
		Day 1 PO (Average of 3 measurements)	3.06 (95% CI 2.44–3.67)	3.16 (95% CI 2.52–3.8)	−0.1 [−0.98, 0.78]
		Day 2 PO (Average of 3 measurements)	2.45 (95% CI 1.81–3.09)	2.36 (95% CI 1.78–2.94)	0.09 [−0.76, 0.94]
		Day 3 PO (Average of 3 measurements)	1.89 (95% CI 1.25–2.52)	1.23 (95% CI 0.74–1.73)	0.66 [−0.14, 1.46]
Dabour et al., 2023 [27] (*n* = 129)	Placebo (*n* = 43)	0 h	0.4 ± 0.88	1.33 ± 1.15	−0.93 [−1.37, −0.49]
		2 h	0.95 ± 1.34	2.14 ± 1.45	−1.19 [−1.79, −0.59]
		4 h	1.2 ± 1.57	2.26 ± 1.53	−1.06 [−1.72, −0.4]
		6 h	1.26 ± 1.36	2.37 ± 1.13	−1.11 [−1.65, −0.57]
		8 h	1.58 ± 1.28	2.81 ± 1.03	−1.23 [−1.73, −0.73]
		12 h	1.95 ± 1.05	2.7 ± 1.2	−0.75 [−1.23, −0.27]
		16 h	2.3 ± 0.9	2.35 ± 1.46	−0.05 [−0.57, 0.47]
		20 h	2.5 ± 1	2.37 ± 1.46	0.13 [−0.41, 0.67]
		24 h	2.5 ± 1.18	1.81 ± 1.28	0.69 [0.15, 1.23]
		Average	1.65 ± 0.7	2.26 ± 0.55	−0.61 [−0.89, −0.33]
	Ketorolac + Orphenadrine (*n* = 43)	0 h	0.4 ± 0.88	0	0.4 [0.14, 0.66]
		2 h	0.95 ± 1.34	0.14 ± 0.5	0.81 [0.37, 1.25]
		4 h	1.2 ± 1.57	0.35 ± 0.8	0.85 [0.31, 1.39]
		6 h	1.26 ± 1.36	0.7 ± 1.1	0.56 [0.02, 1.1]
		8 h	1.58 ± 1.28	1.12 ± 1.2	0.46 [−0.08, 1]
		12 h	1.95 ± 1.05	1.28 ± 0.88	0.67 [0.25, 1.09]
		16 h	2.3 ± 0.9	2.05 ± 0.87	0.25 [−0.13, 0.63]
		20 h	2.5 ± 1	2.56 ± 0.88	−0.06 [−0.46, 0.34]
		24 h	2.5 ± 1.18	2.63 ± 0.85	−0.13 [−0.57, 0.31]
		Average	1.65 ± 0.7	1.2 ± 0.5	0.45 [0.19, 0.71]

Abbreviations: NRS: Numeric Rating Scale; CI: confidence interval; PO: postoperative. Note: Colored cells indicate the direction of effect. Green indicates an effect estimate favoring the fixed-dose combination (Diclofenac/Orphenadrine), while orange indicates an effect estimate favoring the comparator or representing no significant difference.

**Table 8:** Synthesis table for the Morphine Milligram Equivalents consumed in 24 h of the selected studies.

Study	Comparator	Mean Morphine Milligram Equivalents Consumed Intervention 24 h	Mean Morphine Milligram Equivalents Consumed Comparator 24 h	Mean Difference (95% CI Upper, 95% CI Lower)
Danilov et al., 2024 [28] (*n* = 47)	Ketoprofen (*n* = 22)	37.145 ± 15.405	28.665 ± 18.845	8.48 [−1.71, 18.67]
Gombotz et al., 2010 [29] (*n* = 120)	Placebo (*n* = 60)	38.7 ± 21.3	55.9 ± 31.1	−17.2 [−26.84, −7.56]
Eremenko et al., 2022 [30] (*n* = 40)	Morphine monotherapy (*n* = 20)	9.35 ± 4.31	22.6 ± 10.52	−13.25 [−18.39, −8.11]
Borsodi et al., 2008 [31] (*n* = 60)	Tramadol (*n* = 30)	4.51 ± 6.55	17.5 ± 6.5	−12.99 [−16.35, −9.63]
Karelov et al., 2023 [32] (*n* = 48)	Dexketoprofen (*n* = 24)	20.85 ± 5.85	25.85 ± 5.85	−5 [−8.40, −1.60]
Zeiner, 2023 [36] (*n* = 72)	Placebo (*n* = 21)	20.65 ± 12.85	29.5 ± 14.5	−9 [−17.23, −0.47]
	Diclofenac (*n* = 21)	20.65 ± 12.85	28.65 ± 23.75	−8 [−19.77, 3.77]
Yavorovskiy et al., 2023 [35] (*n* = 40)	Ketoprofen (*n* = 20)	0.95(provided total mg per group we divided with 20 to get mean)	2.85 (provided total mg per group, we divided by 20 to get the mean)	−1.9
Dabour et al., 2023 [27] (*n* = 129)	Placebo (*n* = 43)	4.77 ± 5.45 mg	10.7 ± 3.38	−5.93 [−8.63–3.23]
	Ketorolac+Orphenadrine (*n* = 43)	4.77 ± 5.45 mg	1.98 ± 3.47 mg	2.79 [0.07, 5.51]
Gukalov et al., 2023 [38] (*n* = 65)	Morphine only (*n* = 26)	25.50 ± 10.78 (Reported median 25 [19; 33])	29.75 ± 10.20 (Reported median 29 [24; 37])	−4.3625 [−9.64, 0.91] (*p*-value for median 0.085)

Abbreviations: CI: confidence interval; vs.: versus. Note: Colored cells indicate the direction of effect. Green indicates an effect estimate favoring the fixed-dose combination (Diclofenac/Orphenadrine), while orange indicates an effect estimate favoring the comparator or representing no significant difference.

**Table 9:** Synthesis table for the percentage of patients not requiring further opioids.

Study	Comparator	Time Point	%No Opioid Used Intervention	%No Opioid Used Comparator	RR (95% CI Upper, 95% CI Lower)
Dabour et al., 2023 [27] (*n* = 129)	Placebo (*n* = 43)	24 h	51.2% (*N* = 22)	0 (*N* = 0.5)	44 [2.75, 702.97]
	Ketorolac+Orphenadrine (*n* = 43)	24 h	51.2% (*N* = 22)	72.1% (*N* = 31)	0.7 [0.50, 1.00]
Danilov et al., 2024 [28] (*n* = 47)	Ketoprofen (*n* = 22)	24 h	4% (*N* = 1)	31.8% (*N* = 7)	0.13 [0.02, 0.94]
Málek et al., 2004 [33] (*n* = 119)	Placebo (*n* = 40)	24 h	68.6% (*N* = 24)	11.7% (*N* = 5)	5.48 [2.34, 12.83]
	Piroxicam (*n* = 44)	24 h	68.6% (*N* = 24)	52.3% (*N* = 23)	1.31 [0.91, 1.88]
Kuzmina et al., 2023 [39] (*n* = 40)	Ketoprofen (*n* = 20)	24 h	85% (*N* = 17)	70% (*N* = 14)	1.21 [0.86–1.70]
Yavorovskiy et al., 2023 [35] (*n* = 40)	Ketoprofen (*n* = 20) *	24 h	95% (*N* = 19)	75% *N* = 15	1.26 [0.96, 1.66]
Semenkov et al., 2024 [34] (*n* = 40)	Ketoprofen (*n* = 20)	Day 2	60% (*N* = 12)	40% (*N* = 8)	1.5 [0.78, 2.86]
		Day 3	65% (*N* = 13)	25% (*N* = 5)	2.6 [1.14, 5.93]

Note: Colored cells indicate the direction of effect. Green indicates an effect estimate favoring the fixed-dose combination (Diclofenac/Orphenadrine), while orange indicates an effect estimate favoring the comparator or representing no significant difference. * All patients in this study received a continuous background morphine infusion at a dose of 0.1 mg per hour; the data presented reflects the percentage of patients who did not require additional rescue morphine via patient-controlled analgesia (PCA).

**Table 10:** Synthesis table for adverse events reported in the included studies.

System Organ Class/Preferred Term	Study	Comparator	Intervention Group % (N)	Comparator Group % (N)	RR (95% CI Upper, 95% CI Lower)
Gastrointestinal					
Nausea	Eremenko et al. [30]	Morphine monotherapy	5% (*N* = 1)	30% (*N* = 6)	0.16 [0.02, 1.26]
	Semenkov et al. [34]	Ketoprofen	5% (*N* = 1)	40% (*N* = 8)	0.125 [0.017, 0.90]
	Yavorovskiy et al. [35]	Ketoprofen	5% (*N* = 1)	40% (*N* = 8)	0.125 [0.017, 0.90]
	Zeiner et al. [36]	Placebo	26.1% (*N* = 6)	23.8% (*N* = 5)	1.09 [0.39, 3.06]
		Diclofenac	26.1% (*N* = 6)	33.3% (*N* = 7)	0.78 [0.31, 1.95]
	Gombotz et al. [29]	Placebo	31.6% (*N* = 19)	26.6% (*N* = 16)	1.18 [0.67, 2.08]
	Dabour et al. [27]	Placebo	(*N* = 43)	(*N* = 43)	1 [1, 1]
		Ketorolac+Orphenadrine	(*N* = 43)	(*N* = 43)	1 [1, 1]
Vomiting	Eremenko et al. [30]	Morphine monotherapy	5% (*N* = 1)	25% (*N* = 5)	0.2 [0.02, 1.56]
	Semenkov et al. [34]	Ketoprofen	5% (*N* = 1)	20% (*N* = 4)	0.25 [0.03, 2.04]
	Yavorovskiy et al. [35]	Ketoprofen	0% (*N* = 0.5)	5% (*N* = 1)	0.5 [0.01, 14.07]
	Gombotz et al. [29]	Placebo	11.6% (*N* = 7)	13.3% (*N* = 8)	0.875 [0.33, 2.26]
	Dabour et al. [27]	Placebo	14% (*N* = 6)	30.2% (*N* = 13)	0.46 [0.19, 1.10]
		Ketorolac+Orphenadrine	14% (*N* = 6)	4.6% (*N* = 2)	3 [0.64, 14.04]
Dry mouth	Yavorovskiy et al. [35]	Ketoprofen	30% (*N* = 6)	65% (*N* = 13)	0.46 [0.22, 0.96]
Intestinal paresis	Semenkov et al. [34]	Ketoprofen	25% (*N* = 5)	45% (*N* = 9)	0.55 [0.22, 1.36]
	Yavorovskiy et al. [35]	Ketoprofen	5% (*N* = 1)	10% (*N* = 2)	0.5 [0.05, 5.08]
Nervous system disorders					
Drowsiness	Semenkov et al. [34]	Ketoprofen (*n* = 20)	35% (*N* = 7)	60% (*N* = 12)	0.58 [0.29, 1.17]
	Yavorovskiy et al. [35]	Ketoprofen	25% (*N* = 5)	70% (*N* = 14)	0.35 [0.16, 0.80]
Weakness	Semenkov et al. [34]	Ketoprofen (*n* = 20)	40% (*N* = 8)	75(*N* = 15)	0.53 [0.29, 0.96]
Dizziness	Semenkov et al. [34]	Ketoprofen	10% (*N* = 2)	25% (*N* = 5)	0.4 [0.08, 1.82]
General disorders					
Headache	Semenkov et al. [34]	Ketoprofen	15% (*N* = 3)	15% (*N* = 3)	1 [0.23, 4.37]
Decreased rate of diuresis	Semenkov et al. [34]	Ketoprofen	5% (*N* = 1)	10% (*N* = 2)	0.5 [0.05, 5.08]
Fever	Gombotz et al. [29]	Placebo	28% (*N* = 17)	45% (*N* = 27)	0.63 [0.38, 1.02]

Abbreviations: RR: risk ratio; CI: confidence interval; N: number of patients. Note: Colored cells indicate the direction of effect. Green indicates an effect estimate favoring the fixed-dose combination (Diclofenac/Orphenadrine), while orange indicates an effect estimate favoring the comparator or representing no significant difference.

**Table 11:** Summary of findings table using the GRADE method.

Outcome		Participants (Studies)	Risk of Bias	F1 *	F2 *	F3 *	Effect Estimate Range	Certainty of Evidence (GRADE) *	Comments
Analgesic Effect	Pain scores (VAS) at 6 h	195 (4 RCT [28,30,31,32])	Very Serious (−2)	−1	0	−2	MD: −25 to 2.6	⊕◯◯◯ Very Low	The direction of effect was negative in 3 out of 4 studies, favoring FDC. This effect must be interpreted in the context of the opioid-sparing effect found across the wider body of evidence, where studies consistently demonstrated a reduction in the use of narcotic analgesics when FDC was administered.
	Pain scores (VAS) at 24 h	392 (5 RCT [29,30,32,33,36])	Serious (−1)	−1	0	−2	MD: −14 to 2.77	⊕◯◯◯ Very Low	The direction of effect was negative in 4 out of 6 studies, favoring FDC. This effect must be interpreted in the context of the opioid-sparing effect found across the wider body of evidence, where studies consistently demonstrated a reduction in the use of narcotic analgesics when FDC was administered.
	Average pain scores (NRS) at 12 h	238 (2 RCT [27,37])	Very Serious (−2)	−2	0	−2	MD: −0.61 to 0.45	⊕◯◯◯ Very Low	The direction of effect did not consistently favor the FDC since the results were extremely inconsistent.
Opioid sparing effect	Morphine milligrams consumed	554 (9 RCT [27,28,29,30,31,32,35,36,38])	Very Serious (−2)	−1	0	−2	MD: −17.2 to 8.48	⊕◯◯◯ Very Low	The direction of effect was negative in 7 out of 9 studies, favoring FDC. This effect should also be interpreted alongside the consistent finding of lower pain scores reported with the FDC usage.
	% of people without need for opioids	415 (6 RCT [27,28,33,34,35,39])	Very Serious (−2)	−2	0	−1	RR: 44 to 0.12	⊕◯◯◯ Very Low	The direction of effect was above 1 in 5 out of 6 studies favoring FDC. This effect should be interpreted alongside the consistent finding of lower pain scores reported with the FDC usage.
Safety	Incidence of nausea	434 (6 RCT [27,29,30,34,35,36])	Very Serious (−2)	−2	0	−1	RR: 0.12 to 1.19	⊕◯◯◯ Very Low	The direction of effect was below 1 in 4 out of 6 studies, marginally favoring the FDC. However, these results can mask the effect of opioids used in the comparator groups, as the FDC was consistently associated with lower narcotics usage.
	Incidence of vomiting	369 (5 RCT [27,29,30,34,35])	Very Serious (−2)	−2	0	−2	RR: 0.20 to 3	⊕◯◯◯ Very Low	The direction of effect was below 1 in 4 out of 5 studies favoring the FDC. However, these results can mask the effect of opioids used in the comparator groups, as the FDC was consistently associated with lower narcotics usage.

* F1: inconsistency, F2: indirectness, F3: imprecision. GRADE Certainty of Evidence symbols: ⊕⊕⊕⊕ High certainty; ⊕⊕⊕◯ Moderate certainty; ⊕⊕◯◯ Low certainty; ⊕◯◯◯ Very low certainty. Abbreviations: RCT: randomized controlled trial; MD: mean difference; RR: risk ratio; FDC: fixed-dose combination; VAS: Visual Analog Scale; NRS: Numeric Rating Scale.

## Data Availability

No new data were created or analyzed in this study. Data sharing is not applicable to this article.

## Supplementary Materials

The following supporting information can be downloaded at: https://www.mdpi.com/article/10.3390/medicines13020017/s1: S1: PRISMA 2020 checklist; S2: SWiM reporting extension checklist; S3: Full reproducible search strategy for all databases and registries; S4: Detailed PICO characteristics and comprehensive results of included studies. References [27,28,29,30,31,32,33,34,35,36,37,38,39,40] are cited in the Supplementary Material S4.

## Abbreviations

The following abbreviations are used in this manuscript:

AE	Adverse Event
APS	American Pain Society
ASA	American Society of Anesthesiologists
ASRA	American Society of Regional Anesthesia
CI	Confidence Interval
CNS	Central Nervous System
COX	Cyclooxygenase
CRP	C-Reactive Protein
CTIS	Clinical Trials Information System
ERAS	Enhanced Recovery After Surgery
EU	European Union
FDC	Fixed-Dose Combination
GRADE	Grading of Recommendations Assessment, Development and Evaluation
HD	Hospitalization Days
ICU	Intensive Care Unit
IMMPACT	Initiative on Methods, Measurement, and Pain Assessment in Clinical Trials
IMP	Investigational Medicinal Product
IQR	Interquartile Range
MD	Mean Difference
MeD	Median Difference
Miel/MILC	Maximum Inspiratory Capacity of the Lungs
MME	Morphine Milligram Equivalents
NMDA	N-methyl-D-aspartate
NRS	Numeric Rating Scale
NSAID	Non-Steroidal Anti-Inflammatory Drug
ORAEs	Opioid-Related Adverse Events
PICO	Population, Intervention, Comparator, Outcome
PONV	Postoperative Nausea and Vomiting
PRISMA	Preferred Reporting Items for Systematic Reviews and Meta-Analyses
PROSPECT	Procedure-Specific Pain Management
PRS	Physician-Rated Safety
PSS	Patient Satisfaction Score
RASP	Russian Association for the Study of Pain
RCT	Randomized Controlled Trial
RoB2	Risk of Bias 2
RR	Risk Ratio
RSS	Ramsey Sedation Score
SAP	Statistical Analysis Plan
SD	Standard Deviation
SEd	Standard Error of the Difference
SSS	Surgeon Satisfaction Score
SWiM	Synthesis Without Meta-analysis
VAS	Visual Analog Scale
VRS	Verbal Rating Scale
WBF	Weight-Bearing Fraction
WHO	World Health Organization
