# Supplementary material for: The Efficacy of Fixed-Dose Diclofenac and Orphenadrine for Postoperative Pain Management: A Systematic Review
Source: Medicines (Basel). 2026 May 8;13(2):17. doi: 10.3390/medicines13020017 (PMC13214908; doi:10.3390/medicines13020017)
Supplement: Supplementary file 1 [file medicines-13-00017-s001.zip › S2_SWiM reporting extension checklist.pdf]

Synthesis Without Meta-analysis (SWiM) reporting items

The citation for the Synthesis Without Meta-analysis explanation and elaboration article is: Campbell M, McKenzie JE, Sowden A, Katikireddi SV, Brennan SE, Ellis S, Hartmann-Boyce J, Ryan R, Shepperd S, Thomas J, Welch V, Thomson H. Synthesis without meta-analysis (SWiM) in systematic reviews: reporting guideline BMJ 2020;368:l6890 http://dx.doi.org/10.1136/bmj.l6890

**SWiM is intended to complement and be used as an extension to PRISMA**		
**SWiM reporting item**	**Item description**	**Location in thesis**
Methods		
**1** Grouping studies for synthesis	1a) Provide a description of, and rationale for, the groups used in the synthesis (e.g., groupings of populations, interventions, outcomes, study design)	Materials and Methods: Strategy for Data Synthesis
	1b) Detail and provide rationale for any changes made subsequent to the protocol in the groups used in the synthesis	Not applicable
**2** Describe the standardised metric and transformation methods used	Describe the standardised metric for each outcome. Explain why the metric(s) was chosen, and describe any methods used to transform the intervention effects, as reported in the study, to the standardised metric, citing any methodological guidance consulted	Materials and Methods: Effect Measures (MD, MeD, RR; Opioid conversion to MME)
**3** Describe the synthesis methods	Describe and justify the methods used to synthesise the effects for each outcome when it was not possible to undertake a meta-analysis of effect estimates	Materials and Methods: Strategy for Data Synthesis (Descriptive summaries, forest plots without pooling due to heterogeneity)
**4** Criteria used to prioritise results for summary and synthesis	Where applicable, provide the criteria used, with supporting justification, to select the particular studies, or a particular study, for the main synthesis or to draw conclusions from the synthesis (e.g., based on study design, risk of bias assessments, directness in relation to the review question)	Results: Results of the Synthesis (All eligible studies with available data were included; specific timepoints 6h/24h prioritized for visualization)
**SWiM reporting item**	**Item description**	**Location in thesis**

Synthesis Without Meta-analysis (SWiM) reporting items

**5** Investigation of heterogeneity in reported effects	State the method(s) used to examine heterogeneity in reported effects when it was not possible to undertake a meta-analysis of effect estimates and its extensions to investigate heterogeneity	Materials and Methods: Strategy for Data Synthesis (Visual assessment of CI overlap and direction of effect; no statistical test used)
**6** Certainty of evidence	Describe the methods used to assess certainty of the synthesis findings	Materials and Methods: Strategy for Data Synthesis (GRADE methodology)
**7** Data presentation methods	Describe the graphical and tabular methods used to present the effects (e.g., tables, forest plots, harvest plots).<br>Specify key study characteristics (e.g., study design, risk of bias) used to order the studies, in the text and any tables or graphs, clearly referencing the studies included	Materials and Methods: Strategy for Data Synthesis (Tables, Forest plots, Spaghetti plots described)
Results		
**8** Reporting results	For each comparison and outcome, provide a description of the synthesised findings, and the certainty of the findings. Describe the result in language that is consistent with the question the synthesis addresses, and indicate which studies contribute to the synthesis	Results: Results of the Synthesis (Detailed per outcome) and Table 11
Discussion		
**9** Limitations of the synthesis	Report the limitations of the synthesis methods used and/or the groupings used in the synthesis, and how these affect the conclusions that can be drawn in relation to the original review question	Discussion (Limitations of SWiM method and inconsistent timepoints discussed)

PRISMA=Preferred Reporting Items for Systematic Reviews and Meta-Analyses.
*If the information is not provided in the systematic review, give details of where this information is available (e.g., protocol, other published papers (provide citation details), or website (provide the URL)).
