# Supplementary material for: The Efficacy of Fixed-Dose Diclofenac and Orphenadrine for Postoperative Pain Management: A Systematic Review
Source: Medicines (Basel). 2026 May 8;13(2):17. doi: 10.3390/medicines13020017 (PMC13214908; doi:10.3390/medicines13020017)
Supplement: Supplementary file 1 [file medicines-13-00017-s001.zip › S3_Full reproducible search strategy for all databases and registries.pdf]

Search Strategy

- Scopus: ALL ( Diclofenac AND Orphenadrine ) AND ( LIMIT-TO ( SUBJAREA , "MEDI" ) OR LIMIT-TO ( SUBJAREA , "PHAR" ) OR LIMIT-TO ( SUBJAREA , "HEAL" ) OR LIMIT-TO ( SUBJAREA , "DENT" ) OR LIMIT-TO ( SUBJAREA , "NEUR" ) )
- MEDLINE (via PubMed): diclofenac AND orphenadrine
- ResearchGate: diclofenac AND orphenadrine
- ClinicalTrials.gov: diclofenac AND orphenadrine
- WHO International Clinical Trials Registry Platform (ICTRP): diclofenac AND orphenadrine
- EU Clinical Trials Information System (CTIS) / EU Clinical Registry: diclofenac AND orphenadrine
