# Supplementary material for: The Efficacy of Fixed-Dose Diclofenac and Orphenadrine for Postoperative Pain Management: A Systematic Review
Source: Medicines (Basel). 2026 May 8;13(2):17. doi: 10.3390/medicines13020017 (PMC13214908; doi:10.3390/medicines13020017)
Supplement: Supplementary file 1 [file medicines-13-00017-s001.zip › S4_Detailed PICO characteristics and comprehensive results of included studies.pdf]

The characteristics and key findings of the included studies are summarized in the Table below using the PICO (Population, Intervention, Comparator, Outcome) format. To align with the review's objectives, the outcome data for each study have been categorized into Pain, Opioid effect and Safety. Any additional reported measures are listed as Other findings.

Pico table of the included studies

Study	Participants	Intervention	Comparator	Outcome
**Dabour** ***et al*****, 2023**[1]	129 women after mastectomy	Diclofenac + Orphenadrine (150+60)mg/infusion<br>Three times daily + Morphine as needed	• Placebo+ Morphine as needed,<br>• Ketorolac+Orphenadrine (60+60) mg/dose<br>Three times daily<br>+ Morphine as needed	**Pain:** The average NRS pain score during the first 24h was higher on the placebo and Diclofenac+Orphenadrine group compared to the Orphenadrine+Ketorolac group (2.26±.55 vs 1.65±.7 vs 1.2±.5 p<0.002). **Opioid effect:** Ketorolac+Orphenadrine group had more patients requiring no opioids (morphine) than diclofenac + orphenadrine and placebo (72.1% vs 51,2% vs 0%). The differences were statistically significant (p<0.001 for both active treatments vs placebo and p=0.017 for the differences between the active treatment groups). The total dose of the opioid consumed by the Diclofenac+Orphenadrine group was the highest compared to placebo and comparator groups (9.76±3.35mg vs 1.7±3.38 vs 7.08±2.57 respectively). The differences of the Diclofenac+Orphenadrine vs placebo were not statistically significant (p<0.068) while for the ketorolac+orphenadrine it was (p<0.0006).<br>**Safety:** Placebo group had a significantly higher incidence of high post-operative nausea and vomiting (PONV) scores compared to both active treatment groups (0 Vomiting score was for 69.8% of the placebo group vs 86% for the diclofenac+orphenadrine group vs 95.4% for the ketorolac+orphenadrine group, p=0.016), which consequently led to a significantly greater number of patients in that group requiring anti-emetic treatment. No other adverse events were reported.<br>**Other:** During the first 60 minutes post-operatively, sedation levels (RSS) were comparable across all three groups. After this initial hour, however, patients in placebo group experienced a significantly higher frequency of high sedation scores (p<0.05).<br>Both patients and surgeons reported the highest satisfaction with the Ketorolac+Orphenadrine combination.

				• **Patient satisfaction** with post-operative analgesia was significantly higher for the Ketorolac+Orphenadrine infusion (90±7.2) compared to both placebo (70±13) (p<0.001) and the Diclofenac+Orphenadrine infusion (78±12.9) (p<0.001). The Diclofenac+Orphenadrine infusion was also rated as significantly more satisfactory than the placebo (p=0.005).<br>• **Surgeon satisfaction** followed the same pattern. The Ketorolac combination was rated significantly higher (88±6.7) than placebo (76±1.6) (p < 0.001) and the D/O infusion (83±1.6) (p = 0.01). The D/O infusion, in turn, was considered significantly superior to the placebo (p = 0.003).
**Danilov *et al*, 2024**[2]	47 patients after thoracoscopic surgery	Diclofenac + Orphenadrine (75+30) mg/infusion (IV)<br>Twice daily (every 12h) + Trimeperidine as needed	Ketoprofen 100mg/infusion (IV)<br>Twice daily (every 12h) + Trimeperidine as needed	**Pain:** Average VAS scores after 3h, 6h and 9h were not statistically different between the groups (p< 0.37, p<0.72, p<0.23)<br>**Opioid effect:** The amount of opioid (Trimeperidine) consumed after 24h was not statistically different between the two groups (74.29±30.81mg/day for the Diclofenac + Orphenadrine group vs 57.33±33.69 mg/day for the ketoprofen group, p<0.084). However, the ketoprofen group had more patients requiring no further opioids compared to diclofenac+orphenadrine (31.8% vs 4%, p<0.018)<br>**Safety:** The overall safety profiles were comparable between the groups, with no statistically significant differences observed in hemostatic parameters, including platelet count, INR, fibrinogen, and D-dimer. However, FDC demonstrated a potential benefit regarding renal safety; serum cystatin C levels were significantly lower in the intervention group compared to the control after 24 hours of treatment (0.88±0.34 mg/l vs. 1.19±0.44 mg/l; p=0.031)<br>**Other:**<br>• There was no statistically significant difference in cortisol levels between the study group (348.24±0.34 nmol/L) and the control group (402.22± 0.44 nmol/L) (p=0.48). The cortisol levels did decrease in both groups compared to the immediate postoperative period, but this was interpreted as a natural reflection of recovery and decreasing pain over time, not a difference in analgesic efficacy.

				• No statistically significant differences in PaO_2 were found when comparing the groups against each other or when comparing values within the same group (post-operation vs. the next day), with p-values ranging from 0.09 to 0.47
**Gombotz** ***et al*****, 2010** [3]	120 patients after unilateral total hip Arthroplasty	Diclofenac + Orphenadrine (75+30) mg/infusion (IV)<br>Twice daily (every 12h x 1 day)<br>+ Morphine as needed	Placebo<br>+ Morphine as needed	**Pain:** Pain intensities using VAS were comparable between groups at all measured time points. The authors presented this data graphically, and the numerical summary statistics for these time points were not reported.<br>**Opioid effect:** The amount of morphine used was significantly lower for the diclofenac+orphenadrine group compared to the placebo (38.7± 21.3mg vs. 55.9±31.1mg; p<0.0004)) in 24h (~30% less opioid used)<br>**Safety:** The incidence of adverse events were comparable between the groups. No significant safety finding from vital signs or the lab measurement was reported<br>**Other:** Pain intensities using VRS were comparable between groups at all measured time points. The authors presented this data graphically (Figs. 3), and the numerical summary statistics for these time points were not reported
**Eremenko** ***et al*****, 2022**[4]<br>NCT05322603	40 patients after cardiac surgery	Diclofenac + Orphenadrine (75+30) mg/infusion (IV)<br>Once or Twice daily (every 12h) + Morphine as needed	Morphine as needed	**Pain:** The combination group noticed a decrease in VAS score from 41 ±24.27 to 19 ±14.74 in the first hour. After 24h the combination group had a mean VAS score of 14.55±9.5 compared to the morphine monotherapy 27.75±17.43, which was statistically significant (p<0.008)<br>**Opioid effect:** The average dose of morphine used was lower in the diclofenac+orphenadrine group after 4 & 24h compared to the PCA monotherapy (3.45±0.75 vs 6.5±3.25, p=0.001 and 9.35mg vs 22.6mg, p<0.001 respectively).<br>**Safety:** Nausea and vomiting were more common for the morphine group compared to the combination group<br>**Other:** The morphine monotherapy group had lower MIEL values which were statistical significant for all timepoints after the infusion, p<0.05

**Borsodi et al, 2008**[5]	60 patients after breast, testicle and hernia surgery	Diclofenac + Orphenadrine (75+30) mg/infusion (IV)<br>once or twice daily (Second dose after 8h)	Tramadol 50mg (IV), as needed up to 400mg	**Pain:** The mean VAS change for patients who received only the FDC was – 2.5 ± 1.5 and – 3.0 ± 1.6 at rest and after movement respectively. The tramadol group had a mean difference of – 3.7 ± 1.8 at rest and – 3.8 ± 1.7 after movement and patients who received the FDC+Tramadol had a mean change of – 3.0 ± 1.7 at rest and – 3.4 ± 1.8 after movement. All these changes were for the first 6 hours. The authors presented p values for the mean change which was statistically significant for all values (p<0.001).<br>**Opioid effect:** The opioid consumption was measured over this entire 74 patient-days (1.2-day average period) Patients who got the FDC and still required supplemental analgesia consumed less tramadol 61.5±21.9, whereas patients receiving only tramadol required 87.5 ± 32.5 mg (p<0.01 for the difference).<br>**Safety:** Physician-rated tolerability showed no difference between the groups. Physician-rated safety was considered "very good" or "good" in all three groups. 3 AEs were reported, 2 nausea and 1 vomiting but the authors did not report the incidence between the groups.<br>**Other:** Regarding the effectiveness, no "unsatisfactory, weak or ineffective" responses were reported for any group. The FDC received the best effectiveness rating from both patients and doctors in a statistical comparison. Regarding practicality, nurse evaluations rated the diclofenac-orphenadrine group as good in 12 cases, very good in 5, and poor in 2, while the tramadol group was rated 73% good and 27% average, and the third group 72% good. For time-saving, the diclofenac-orphenadrine group was rated 79% good, 2 very good, and 2 average; the tramadol group was 53% good and 47% average; and the third group received 8 good and 3 very good ratings.
**Karelov** ***et al*****, 2023**[6]	48 patients after abdominal and vertebral surgeries	Diclofenac + Orphenadrine (75+30) mg/infusion (IV)<br>Once before surgery and 12h after + trimeperidine 20mg as needed +	Dexketoprofen 50mg IV<br>Once after awakening and 1 after 8h + trimeperidine 20mg as needed+ paracetamol as needed	**Pain:** The FDC group had less average VAS scores compared to the dexketoprofen group at the awakening (3±0.6 vs 3.5±0.7, p<0.05) and after 12h (4.1 vs 4.9, p<0.05). For the other timepoints after 3h, 6h and 24h both groups had comparable average VAS scores. The dexketorprofen group used more rescue medication (paracetamol) (1000.0±722.3 vs 541.7± 509.0)<br>**Opioid effect:** After 24h the FDC required less trimeperidine than the dexketoprofen group (41.7±11.7 vs 51.7±11.7, p<0.05).

		paracetamol as needed		Other: The FDC group after 24 h required less paracetamol than the comparator (541.7±509.0 vs1000.0±722.3, p<0.05).
**Málek** ***et al*****, 2004**[7]	119 Patients after knee arthroscopy	Diclofenac + Orphenadrine (75+30) mg/infusion (IV)<br>Once daily + pethidine as needed	• Placebo + pethidine as needed,<br>• Piroxicam 20 mg/ml IV + pethidine as needed	**Pain:** The average VAS score after 24h was lower for the FDC compared to placebo and piroxicam (FDC 1.5 vs. Placebo 2.9 p < 0.05, Piroxicam 2.4 vs. Placebo 2.9 p < 0.05)<br>**Opioid effect**: The opioid sparing effect, the percent of people not requiring further analgesic, was higher for the FDC compared to placebo and piroxicam (68.6% vs 11.7% vs 52.3% , p<0.001)<br>**Safety:** FDC had fewer adverse events that piroxicam and placebo, p<0.05
**Semenkov** ***et al*****, 2024**[8]	40 Patients after abdominal oncologic surgery	Diclofenac + Orphenadrine (75+30) mg/infusion (IV)<br>Twice daily x 2 days + Tramadol as needed	Ketoprofen 200mg/day IV + Tramadol as needed	**Pain:** The diclofenac-orphenadrine group (Group H) showed a more pronounced analgesic effect compared to the ketoprofen group (Group K). While both groups started with similar pain levels, with 80% of patients in each group experiencing moderate pain (VAS 41-70 mm), their paths diverged. On the second post-operative day, 60% of Group K patients had moderate pain , compared to only 30% in Group H. This gap widened by the third day, with 75% of Group K patients reporting moderate pain , while the rate in Group H fell to 15%. By the fourth day, 100% of patients in Group H experienced only low-intensity pain.<br>**Opioid effect:** A greater percentage of patients in the FDC group did not require rescue tramadol compared to the ketoprofen group on both the 2nd day (60% vs 40%) and the 3rd day (65% vs 25%)<br>**Safety:** In the FDC group nausea, drowsiness and weakness were less common that in the ketoprofen group (p<0.05). Average creatin and CRP values did not differ statistically between groups. Clavien-Dindo complications were also comparable.<br>**Other:** The average length of hospital stay was comparable between groups.
**Yavorovskiy** ***et al*****, 2023**[9]	40 Patients after thoracic surgery	Diclofenac + Orphenadrine (75+30) mg/infusion (IV)	Ketoprofen 100mg IV<br>Once or twice daily + morphine as needed	**Pain:** The median VAS scores were lower for the FDC group after 1,2,6,8,10,12,18,24 (p<0.001) except for the 4h hour timepoint were it was comparable.<br>**Opioid effect:** The total amount of morphine used in the FDC group was lower than that of the ketoprofen group (19mg vs 57mg)

		Once of twice daily + morphine as needed		**Safety:** The FDC group had less incidence of adverse events compared to ketoprofen. The authors assessed the potential nephrotoxicity of both arms measuring the creatine levels, which confirmed the absence of kidney damage in both groups. The average diuresis remained the same for both groups.<br>**Other:** Patients in the FDC group had higher MIEL scores than the Ketoprofen group at most timepoints, p<0.05.
**Zeiner, 2023**[10]<br>NCT03493490	72 patients undergoing elective cruciate<br>ligament surgery	Diclofenac + Orphenadrine (75+30) mg/infusion (IV)<br>Three times daily x 1 day + hydromorphone as needed	• Diclofenac 75mg IV three times daily + hydromorphone as needed<br>• Placebo + hydromorphone as needed	**Pain:** Mean VAS scores between the treatment arms were not statistically significant after 30min, 120min and 24h<br>**Opioid effect:** The amount of hydromorphone consumed between the treatment arms was not statistically significant 24h post surgery (5.90mg (SD=2.90) placebo, 5.73mg (SD=4.75) diclofenac, 4.13mg (SD=2.57) FDC). In a post-hoc analysis combining both the FDC and the diclofenac group showed a statistically significant decrease in opioid consumption compared to placebo, (4.34mg vs 5.89 mg, p=0.049).<br>**Safety:** No severe adverse events were reported and nausea cases were comparable across groups.<br>**Other:** No difference between groups was found for the delirium score.
**Tomic, 2022**[11]	109 patients undergoing orthognathic surgery	Ibuprofen 600mg IV<br>Twice daily + piritramide + paracetamol as needed	Diclofenac + Orphenadrine (75+30) mg/infusion (IV)<br>Twice daily + piritramide + paracetamol as needed	**Pain:** The difference for the NRS scores over the 3 days study period between the treatments was not statistically significant when analyzing all patients as per the primary endpoint (p<0.352). Groups had a mean NRS score in the third day of 1.89 (1.25–2.52) and 1.23 (0.74–1.73) for FDC and Ibuprofen groups respectively. After doing a subgroup analysis authors concluded that for the BIMAX subgroup Ibuprofen was superior (2.73 vs.1.23 , p<0.015) on the 3rd day.<br>**Opioid effect**: No significant difference of piritramide intake was found between the groups (n=41 for the FDC , n =31 for the Ibuprofen).<br>**Safety:** No AEs were reported.<br>**Other:** The study found no significant differences between the FDC group and the ibuprofen group in length of stay (6.21 vs. 6.16 days), or the rate of chin osteotomies. However, the ibuprofen group required significantly more rescue acetaminophen for pain (n=46 vs n=78, p = 0.006).

**Gukalov** ***et al*****, 2023**[12]	65 patients after hip arthroplasty	Diclofenac + Orphenadrine (75+30) mg/infusion (IV)<br><br>Once or twice daily + morphine as needed	Morphine as needed	**Pain:** VAS scores (median) were comparable between FDC and the comparator group after 2h (5 [4-6] vs 5[4-6], p= 0.813) and 12h [2-3]vs 3[2-4], p= 0.071). After 24h FDC had lower VAS scores (2 [2-3 vs 3 [2; 3], p= 0.057].<br><br>**Opioid effect:** Morphine doses between groups were not statistically significant (25 mg [19- 33] in the FDC vs 29 [24- 37] mg, p=0.085)<br><br>**Safety:** The incidence of adverse events was comparable between groups and included nausea, vomiting and itching (15% in both groups).
**Kuzmina** ***et al*** **2023** [13]	40 patients after knee arthroplasty	Diclofenac + Orphenadrine (75+30) mg/infusion (IV)<br><br>Twice daily for 2 days + tramadol + trimeperidine as needed	Ketoprofen 100mg IV<br><br>Twice daily for 2 days + tramadol + trimeperidine as needed	**Pain:** The FDC group at 24h had a median of 2.5 (2-3) whereas the comparator had 4 (3-5), which was statistically significant, p<0.006. At 48 h FDC was still superior having a median of 2 (2-3) vs 3 (2.8-4), p=0.021<br><br>**Opioid effect:** Tramadol usage was comparable between groups and not statistical significant (85% did not use tramadol in the FDC vs 70% in the comparator, p=0.256). The authors do not report if trimeperidine was used by any subject.<br><br>**Safety:** No adverse events were reported in the study<br><br>**Other:** Restoration of the weight-bearing capacity of the FDC was higher than the ketoprofen group after 24h (85%vs65%) and after 48h (95%vs 85%), which was not statistically significant, p=0.144 and p=0.256 respectively.
**Lukonina et al, 2024**[14]	52 cancer patients after spinal surgery	Diclofenac + Orphenadrine (75+30) mg/infusion (IV)<br><br>Once intra-operative 30 min before the end then once (whole infusion) or twice (half the infusion) daily for 2 days + tramadol as needed	• Tramadol 100mg 30min before the end of surgery and on demand + morphine if required<br>• Ibuprofen 800 mg IV 30min before end of surgery and 800 mg once or 400mg twice daily for 2 days + tramadol as needed+ morphine if required	**Pain:** Significant differences were found in pain intensity in the morning of postoperative day 2 (24h) between FDC (Median=10 (0-20), Ibuprofen Median = 20 (10-30) and tramadol monotherapy Me=40 (30-48), p<0.03. No difference was found on the other timepoints (1h,3h, 12h)<br><br>**Opioid effect:** The duration of opioid therapy was significantly shorter in the non-opioid groups (Group I and Group D: 6 postoperative days each) compared to the control group (Group K: 7 days) (p=0.013)<br><br>**Safety:** No adverse events were reported.<br><br>**Other:** Patient and physician satisfaction with pain relief was high ("good" or "excellent") and did not differ between groups at one hour

		+ morphine if required		post-administration. On the first postoperative day, drainage volume was not significantly different between the FDC and the tramadol group (p=0.450), and no vasopressor support was needed. Additionally, a statistically significant decrease in subcutaneous cortisol was recorded in the ibuprofen group.

Abbreviations*:* FDC: Fixed-Dose Combination; IV: Intravenous; NRS: Numeric Rating Scale; VAS: Visual Analogue Scale; PONV: Postoperative Nausea and Vomiting; RSS: Ramsey Sedation Score; SD: Standard Deviation; D/O: Diclofenac/Orphenadrine.

References

1. Dabour, Y.S.; Elnoury, A.S.; Daoud, A.A. Twenty-Four-Hour Postoperative Orphenadrine and Ketorolac Infusion Efficiently Precedes Orphenadrine-Diclofenac Infusion as an Opioid-Sparing Analgesic Modality after Mastectomy. *Egypt. J. Anaesth.* **2023**, *39*, 706–714. https://doi.org/10.1080/11101849.2023.2247231.
2. Danilov, M.S.; Simutis, I.S.; Salygina, D.S.; Polovtsev, E.G.; Syrovatskiy, A.A.; Bogatikov, A.A.; Fedorov, M.E. Advisability of NSAID Combined with Central Muscle Relaxant for Pain Syndrome after Thoracoscopic Surgery. *Russ. J. Anesthesiol. Reanimatol.* **2024**, *5*, 82–87. https://doi.org/10.17116/anaesthesiology202405182.
3. Gombotz, H.; Lochner, R.; Sigl, R.; Blasl, J.; Herzer, G.; Trimmel, H. Opiate Sparing Effect of Fixed Combination of Diclophenac and Orphenadrine after Unilateral Total Hip Arthroplasty: A Double-Blind, Randomized, Placebo-Controlled, Multi-Centre Clinical Trial. *Wien. Med. Wochenschr.* **2010**, *160*, 526–534. https://doi.org/10.1007/s10354-010-0829-7.
4. Eremenko, A.A.; Sorokina, L.S.; Ryabova, D.V.; Urbanov, A.V. Analgesic and Opioid-Sparing Effects of a Fixed Combination of Diclofenac and Orphenadrine in the Early Postoperative Period in Cardiac Surgery Patients. *S.S. Korsakov J. Neurol. Psychiatry* **2022**, *122*, 109. https://doi.org/10.17116/jnevro2022122101109.
5. Borsodi, M.; Nagy, E.; Darvas, K. Diclofenac/Orphenadrin as a Combined Analgetic in Post-Operative Relief of Pain. *Orvosi Hetilap* **2008**, *149*, 1847–1852. https://doi.org/10.1556/oh.2008.28419.
6. Karelov, A.E.; Khmara, V.M.; Orozaliev, I.E.; Kellarev, M.V.; Petrosyan, T.G.; Karelov, D.A.; Zabolotskii, D.V. Comparative Assessment of Analgesic Efficacy of Diclofenac and Orfenadrine Combination in Abdominal and Vertebral Surgery. *Russ. J. Anesthesiol. Reanimatol.* **2023**, *5*, 52–57. https://doi.org/10.17116/anaesthesiology202305152.
7. Málek, J.; Nedělová, I.; Lopourová, M.; Stefan, M.; Kostál, R. Diclofenac 75mg. and 30 mg. orfenadine (Neodolpasse) versus placebo and piroxicam in postoperative analgesia after arthroscopy. *Acta Chir. Orthop. Traumatol. Cechoslov.* **2004**, *71*, 80–83.
8. Semenkov, A.V.; Skugarev, A.L.; Tulskih, D.A.; Khitrov, N.V.; Yavorovskaya, D.A.; Sergeev, O.S. Pain Relief after Abdominal Oncologic Surgery. Evaluation of the Effectiveness and Safety of a Fixed Combination of Diclofenac and Orphenadrine. *Pirogov Russ. J. Surg.* **2024**, *9*, 38–50. https://doi.org/10.17116/hirurgia202409138.

9. Yavorovskiy, A.G.; Nogtev, P.V.; Ovechkin, A.M.; Bagdasarov, P.S.; Polukhin, N.V.; Sergeev, O.S.; Aliev, V.A.; Zolotova, E.N.; Yavorovskaya, D.A. Postoperative Analgesia with a Fixed Combination of Diclofenac and Orphenadrine in Thoracic Surgery. *Russ. J. Anesthesiol. Reanimatol.* **2023**, *4*, 81–87. https://doi.org/10.17116/anaesthesiology202304181.
10. Zeiner, S.; Haider, T.; Zotti, O.; Thüringer, K.; Höbart, P.; Kimberger, O.; Knolle, E. Intravenous Diclofenac and Orphenadrine for the Treatment of Postoperative Pain after Remifentanil-Based Anesthesia: A Double-Blinded, Randomized, Placebo-Controlled Study. *Wien. Klin. Wochenschr.* **2023**, *135*, 67–74. https://doi.org/10.1007/s00508-022-02131-x.
11. Tomic, J.; Wallner, J.; Mischak, I.; Sendlhofer, G.; Zemann, W.; Schanbacher, M.; Hassanzadeh, H.; Sandner-Kiesling, A.; Payer, M.; Zrnc, T.A. Intravenous Ibuprofen versus Diclofenac plus Orphenadrine in Orthognathic Surgery: A Prospective, Randomized, Double-Blind, Controlled Clinical Study. *Clin. Oral. Investig.* **2022**, *26*, 4117–4125. https://doi.org/10.1007/s00784-022-04381-5.
12. Gukalov, A.A.; Klypa, T.V.; Mandel', I.A.; Minets, A.I. The Use of a Fixed Combination of Diclofenac and Orphenadrine for Postoperative Pain Relief in Orthopedic Patients. *Pirogov Russ. J. Surg.* **2023**, *4*, 49–54. https://doi.org/10.17116/hirurgia202304149.
13. Kuzmina, M.V.; Shlyk, I.V.; Panafidina, V.A.; Kozhevin, A.A.; Polushin, Y.S.; Krivov, V.O. The Use of a Combination of Diclofenac and Orphenadrine for Analgesia in Knee Replacement. *Messenger Anesthesiol. Resusc.* **2023**, *20*, 48–53. https://doi.org/10.24884/2078-5658-2023-20-5-48-53.
14. Lukonina, T.D.; Денисовна, Л.Т.; Khoronenko, V.E.; Эдуардовна, Х.В.; Abuzarova, G.R.; Рафаиловна, А.Г.; Alekseeva, G.S.; Сергеевна, А.Г.; Buharov, A.V.; Викторович, Б.А. Potential of modern nonopioid analgesics in the prevention and treatment of pain syndrome in decompressive stabilizing spine surgeries. *Reg. Anesth. Acute Pain Manag.* **2024**, *18*, 115–131. https://doi.org/10.17816/RA628829.
